# Ras hyperactivation versus overexpression: Lessons from Ras dynamics in *Candida albicans*

**DOI:** 10.1038/s41598-018-23187-8

**Published:** 2018-03-27

**Authors:** Vavilala A. Pratyusha, Guiliana Soraya Victoria, Mohammad Firoz Khan, Dominic T. Haokip, Bhawna Yadav, Nibedita Pal, Subhash Chandra Sethi, Priyanka Jain, Sneh Lata Singh, Sobhan Sen, Sneha Sudha Komath

**Affiliations:** 1 School of Life Sciences, Jawaharlal Nehru University, New Delhi, 110067 India; 2 School of Physical Sciences, Jawaharlal Nehru University, New Delhi, 110067 India; 3 Present Address: Post-doctoral fellow, Cell Biology of Mammalian Neurogenesis, Institut Curie, Paris, France; 4 Present Address: Biochemistry and Molecular Pharmacology Department, University of Massachusetts Medical School 55 Lake Ave N, Worcester, 01655 MA USA; 5 Present Address: The Aberdeen Fungal Group, School of Medicine, Medical Science and Nutrition, Institute of Medical Sciences, University of Aberdeen, Aberdeen, AB252ZD UK; 6 Present Address: Department of Chemistry, University of Michigan, Ann Arbor, MI 48109-1055 USA; 7 Present Address: Department of Chemistry, Indian Institute of Technology, Delhi, India

**Keywords:** Fluorescence spectroscopy, Membrane biophysics, Cell signalling, Fungal biology

## Abstract

Ras signaling in response to environmental cues is critical for cellular morphogenesis in eukaryotes. This signaling is tightly regulated and its activation involves multiple players. Sometimes Ras signaling may be hyperactivated. In *C. albicans*, a human pathogenic fungus, we demonstrate that dynamics of hyperactivated Ras1 (Ras1G13V or Ras1 in Hsp90 deficient strains) can be reliably differentiated from that of normal Ras1 at (near) single molecule level using fluorescence correlation spectroscopy (FCS). Ras1 hyperactivation results in significantly slower dynamics due to actin polymerization. Activating actin polymerization by jasplakinolide can produce hyperactivated Ras1 dynamics. In a sterol-deficient hyperfilamentous GPI mutant of *C. albicans* too, Ras1 hyperactivation results from Hsp90 downregulation and causes actin polymerization. Hyperactivated Ras1 co-localizes with G-actin at the plasma membrane rather than with F-actin. Depolymerizing actin with cytochalasin D results in faster Ras1 dynamics in these and other strains that show Ras1 hyperactivation. Further, ergosterol does not influence Ras1 dynamics.

## Introduction

Ras is an oncogene whose hyperactivation plays a role in many types of cancers^1^. Besides controlling differentiation and morphogenesis, Ras signaling pathway in eukaryotes regulates cellular response to external stimuli^2^. Recent studies have helped delineate the dynamics of different forms of Ras, their dependence on membrane composition/organization, their interactions and specific compartments for signaling in the mammalian system^3–5^, which have helped explain the effect of Ras activation/inactivation on mammalian cellular processes.

In yeast and fungi, the Ras signaling cascade determines gene expression while attenuating growth as well as morphogenetic transformations in response to different environmental conditions^6^. In *C. albicans*, a major human pathogenic fungus, such transformations including those from yeast to hyphal forms are extremely important for infection and virulence. Several hyphae-specific proteins are virulence factors that not only play a role in host recognition and tissue invasion but also actively participate in evasion of host defense mechanisms^7^.

Ras in *C. albicans* is farnesylated and palmitoylated before being reversibly associated with the inner layer of the plasma membrane. Regulated proteolytic cleavage controls the extent of membrane associated Ras available for participating in the signaling cascade^8^. Activation of signaling requires Ras to cycle between an inactive, GDP-bound form and its active GTP-bound form. Alterations in the association of Ras with members of the signaling cascade can also alter its level of activation. For example, a mutation in Ras that inhibits its association with Ira2, a GTPase activating protein (GAP), prevents its transition to the inactive form, causing Ras to be constitutively activated^9^. Another common mechanism for activating Ras in *C. albicans* involves reducing levels of the inhibitory heat shock protein, Hsp90, triggered by growth at 37 °C^10^. Under ambient conditions, Hsp90 in association with its co-chaperone, Sgt1, inhibits the interaction of GTP-bound Ras with the adenylyl cyclase, Cyr1, which inhibits the downstream cAMP/PKA signaling pathway and promotes conversion of Ras(GTP) back to Ras(GDP)^11^. A lifting of this inhibition, by depleting available levels of Hsp90, permits the organism to rapidly transform from yeast to hyphae form for invasive colonization of the host^10,11^. Overexpression of Ras can also result in heightened cAMP/PKA signaling. Are these activated states equivalent?

Using *C. albicans* as our model organism, we set out to explore whether some of these mechanisms of activation could be reliably tracked and distinguished from others. Fluorescence correlation spectroscopy (FCS) was used as the primary tool of analysis since it permits us to monitor (translational) diffusion of a single (or a few) probe molecule(s) over a relatively long distance and monitor diffusion events that occur in time scales ranging from microseconds to seconds. While some studies have successfully employed FCS to study protein dynamics in bacteria and baker’s yeast^12–14^ no study has so far explored the use of FCS in *C. albicans*.

We begin by exploring the difference in diffusion dynamics of membrane bound Ras, at near single molecule level, when it is overexpressed versus when it is constitutively activated via a G13V mutation^6^. We demonstrate that the latter is a hyperactivated state that involves actin polymerization while the former is not. Ras signaling in mutants deficient in Hsp90 also involves actin activation. Using a hyperfilamentous mutant of *C. albicans* we also demonstrate that the mechanism of Ras hyperactivation in this strain may be attributed to Hsp90 downregulation and actin polymerization. In the end, we propose a probable mechanism for how Ras hyperactivation and actin polymerization are linked.

## Results

### Expressing fluorescently tagged Ras1 in wild type *C. albicans* strains

**Figure 1:**
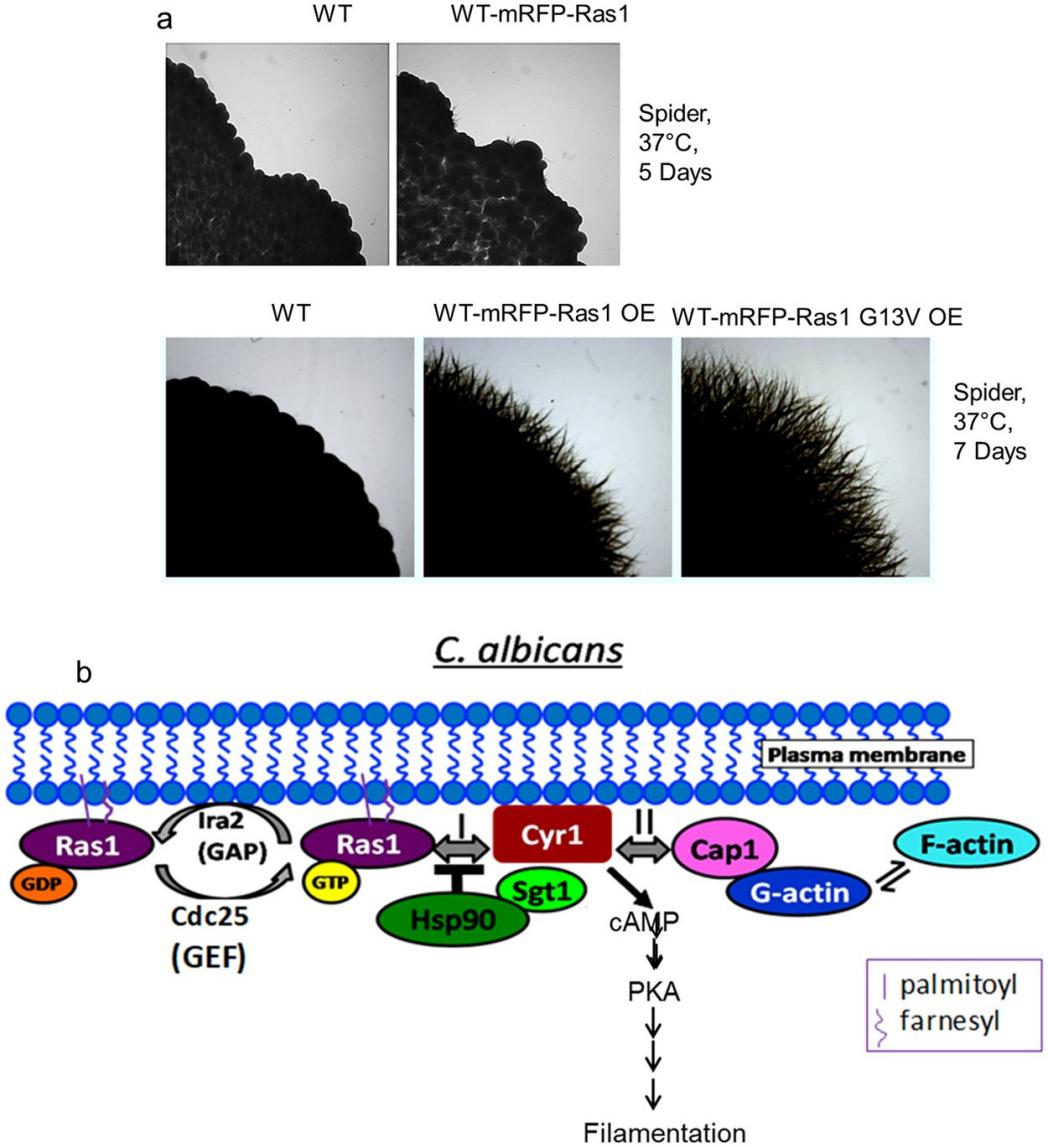
Dynamics of Ras1 in its wild type (WT), overexpressed and hyperactivated states in *C. albicans*. (**a**) Filamentation pattern of mRFP tagged Ras1 in the *C.albicans* wild type (WT) as well as in Ras1 and Ras1G13V overexpression (OE) strains. Strains overexpressing Ras1 or Ras1G13V are hyperfilamentous. The experiment was repeated thrice and representative images are shown. (**b**) Ras signaling in *C. albicans via* the cAMP-PKA pathway. In yeast cells, Ras1 cycles between an inactive GDP-bound form and an active GTP-bound form with the help of Cdc25, a GEF, and Ira2, a GAP. Ras1(GTP) binds to its effector, Cyr1, and activates it to produce cAMP (path I), thereby kick-starting the PKA dependent signaling pathway. However, in yeast cells, the interaction between Ras1(GTP) and Cyr1 is inhibited by Hsp90 and its co-chaperone Sgt1, resulting in only a basal level of cAMP-dependent PKA signaling. Exposure to physiological temperature or other hyphae inducing conditions such as serum are necessary for this inhibition to be lifted and for Cyr1 to produce cAMP, stimulating the PKA signaling pathway and generating hyphae. Upon receiving specific signals, such as CO_2_ or HCO_3_^−^, Cyr1 can also directly produce cAMP in a Ras-independent manner via its interaction with Cap1 which in turn interacts with G-actin (path II). The polymerization of actin is triggered by the binding of G-actin to the Cyr1-Cap1 complex. However, no evidence till date suggests that both pathways can operate simultaneously.

To monitor Ras dynamics using FCS, the protein needs to be fluorescently tagged. *C. albicans* possesses two Ras proteins, Ras1 and Ras2. Of these, Ras1, is majorly involved in hyphal morphogenesis^15^. Hence, using its native promoter Ras1 was tagged at its N-terminus with yEmRFP in BWP17 cells (hereafter referred to as mRFP). This allowed us to track dynamics of wild type Ras1 at its normal expression levels. The protein, mRFP-Ras1 predominantly localized to the plasma membrane (Supplementary Fig. 1a). The low fluorescence intensity confirmed that mRFP-Ras1 was not expressed at high levels and was suitable for FCS measurements at (near) single molecule level. The tagging did not affect Ras signaling and the strain showed a filamentation phenotype very similar to that of control BWP17 cells (Fig. 1a). For overexpressing Ras1 or its constitutively active variant, Ras1G13V, the strong *ADH*1 promoter was used to introduce a copy of these genes in the WT at the *RPS1* locus^6^. Both strains showed hyperfilamentation phenotypes as expected (Fig. 1a). The localization of Ras1 in both cases was predominantly in the plasma membrane (Supplementary Fig. 1b).

### Hyperactivation of Ras1 results in a significant reduction in its dynamics while overexpression does not

Normally, in yeast cells, Ras1 is expected to be predominantly present bound to GDP, as Ras1(GDP), its OFF state^6^ (Fig. 1b). Upon receiving a stimulus for activation, Ras1(GDP) would convert to Ras1(GTP), its ON state, with the help of guanine nucleotide exchange factors (GEFs). This exposes the effector binding loop of Ras1 and it can bind to Cyr1, its downstream effector, to turn on the signaling cascade (path I in Fig. 1b). The Ras1-Cyr1 interaction is inhibited by Hsp90 in association with Sgt1, its co-chaperone. The conversion of Ras1(GTP) back to Ras1(GDP), occurs via GTPase activating proteins (GAPs) that stimulate hydrolysis of GTP. In *C. albicans*, the only Ras1-specific GEF identified so far is Cdc25^16^ and GAP is Ira2^6^.

**Figure 2:**
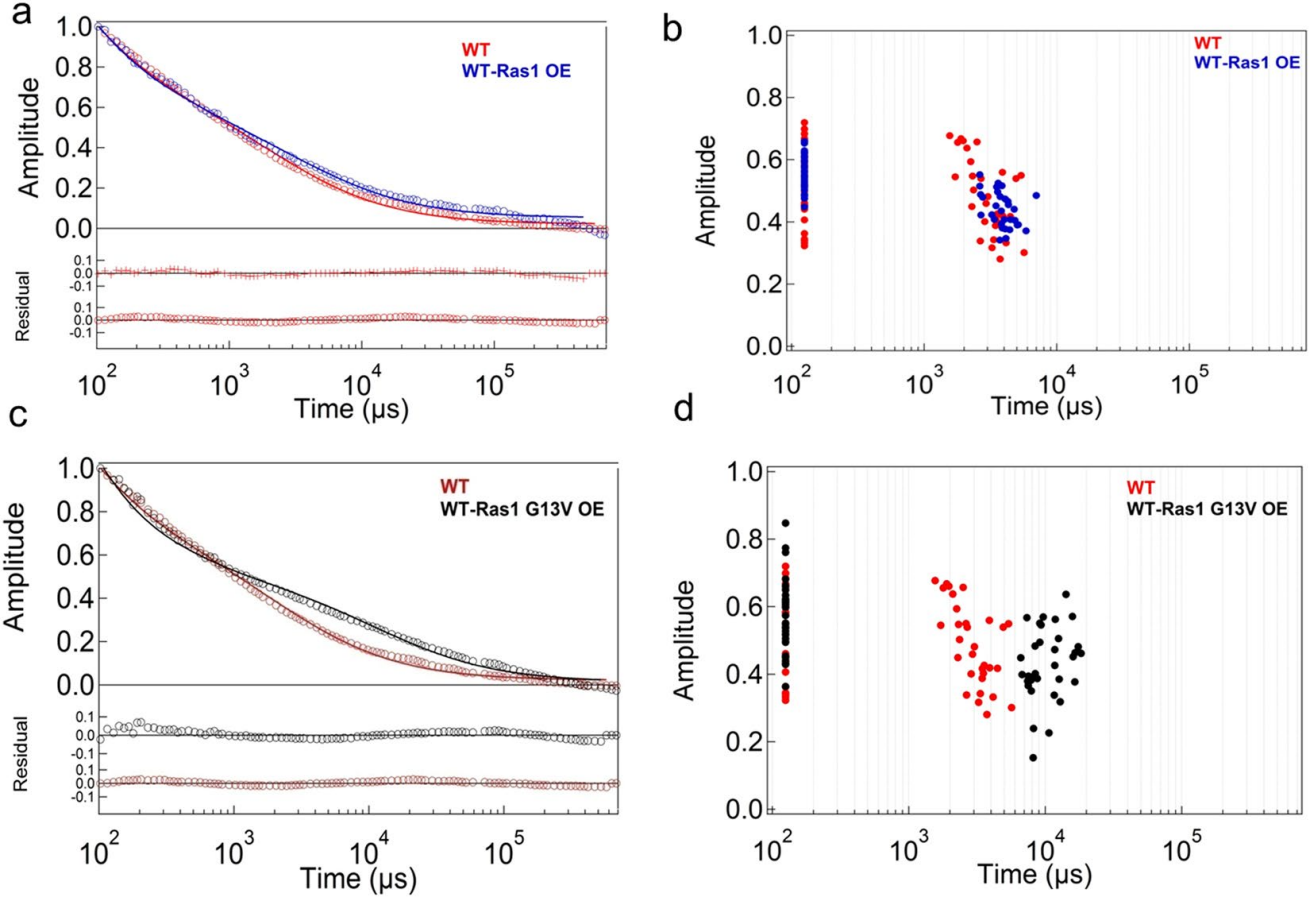
Slower dynamics is seen upon constitutive activation of Ras1. Average fluorescence autocorrelation, *G*(τ), curves (**a**,**c**) and plot of amplitude versus diffusion times obtained in individual cells (**b**,**d**) for mRFP-Ras1 in wild type (WT) as well as overexpressed states of Ras1 and Ras1G13V. The data was collected for 35–40 cells in each case.

The diffusion dynamics of fluorescently tagged Ras1 (mRFP-Ras1) expressed in the wild type (WT) strain using its native promoter or overexpressed using the *ADH1* promoter was monitored by FCS. The fluorescence auto-correlation data in both cases could be best fit to a two-dimensional two-component (2D2C) diffusion model, multiplied with an exponential component contributing to the flickering process of mRFP (see equation 5 in Materials and Methods; Fig. 2a). A two-dimensional (2D) reference frame was chosen since it best represented the diffusion model for Ras1 dynamics in the inner leaflet of the plasma membrane. Besides the flickering component (*τ*_*T*_), which was set to 65 μs^17^, there were two diffusion components (*τ*_*D1*_ and *τ*_*D*2_) that were hypothesized to be contributing to the diffusion times for Ras1. The reason for using a 2D2C model is that diffraction limited laser spot at the sample position would encompass regions of both membrane and the cytoplasm (see Materials and Methods). Thus, the contribution of unhindered diffusion of Ras1 in cytoplasm is represented by *τ*_*D1*,_ while the diffusion time of membrane localized form of Ras1 is represented by *τ*_*D2*_. It should be noted that the values of *τ*_*D2*_ are significantly different from the unhindered diffusion of Ras1, which are altered drastically in the mutants – suggesting changes in diffusion of membrane bound Ras1 only. Engleborgh’s lab estimated the free diffusion time for mRFP as 39 ± 17 μs (expressed as a fusion with eGFP) in the cytoplasm of human HeLaP4 cells, along with a flickering time-constant of 63 μs^17^. Therefore, the diffusion time for free mRFP in yeast cells is expected to be small. To obtain a rough estimate of this, we expressed a cytosolic form of mRFP in *C. albicans* wild type cells and measured its dynamics after rupturing the cells with 0.1% Triton X-100. We found that free mRFP gave a diffusion time of ~106–133 μs (Supplementary Fig. 2a). Based on this, *τ*_*D1*_ was fixed at 125 μs in all the data sets. Keeping this component free did not significantly alter the fitting of the data, but keeping it fixed allowed us to focus exclusively on the second component. The second component yielded a much longer diffusion time (*τ*_*D2*_), which was kept free in all the fitting analyses to observe the differences in relative mobility of Ras1 within the membranes of different cells. In our model this second component corresponds to membrane-bound Ras1 whose mobility in the wild type cells is hindered due to its localization in the membrane. In cells where Ras1 is hyperactivated, we suggest that this component is further hindered by interaction of Ras1 with other proteins/downstream effectors.

Figure 2a compares the averaged auto-correlation curves of mRFP-Ras1 in wild type (WT) cells expressed at endogenous levels versus that of overexpressed mRFP-Ras1, along with fits using equation 5 (the average of 30–45 correlation curves, measured in as many cells for a particular strain, was used for final comparison of average Ras1 diffusion dynamics in Table 1). The distributions of the two diffusion components extracted from auto-correlation curves measured in several yeast cells of the two strains are shown in Fig. 2b. As can be seen from Fig. 2a and b, overexpressing Ras1 did not significantly alter the dynamics of Ras1 in *C. albicans* cells vis-à-vis endogenous Ras1 (Table 1). Overexpressing constitutively activated Ras1G13V, however, resulted in significantly slower dynamics (Fig. 2c and d; Table 1). We also obtained comparable results when these Ras1 variants were expressed in the *Caras1* null, where no endogenous Ras1 is present (Supplementary Fig. 3; Table I). Is this also true for other hyperactivated states of Ras1?

**Table 1:** Average diffusion times for yEmRFP-Ras1 and Nile red in the yeast form of the wild type and conditional null *Cagpi19* strains as measured by FCS.

yEmRFP-Ras1	
Strain and experimental conditions	Average Diffusion Time (*τ*_*D2*_) (ms)
Wild type (WT)	2.63 ± 0.24
WT-Ras1 OE (overexpressed)	3.97 ± 0.40
WT-Ras1G13V OE (overexpressed)	12.48 ± 1.68
*Caras1* null-Ras1 OE	2.24 ± 0.50
*Caras1* null-Ras1G13V OE	10.57 ± 0.89
WT + geldanamycin	9.84 ± 0.23
*Cahsp90* null	17.13 ± 0.47
WT + JasP	14.53 ± 2.02
WT-Ras1G13V + CytoD	3.1 ± 0.19
*Cahsp90* null + CytoD	2.77 ± 0.79
*Cagpi19* null	15.21 ± 0.48
*Cagpi19* null + tamoxifen	4.42 ± 0.43
*Cagpi19* null + CytoD	4.35 ± 0.68
*Cagpi19* null + LatB	6.18 ± 0.28
WT + βCD	4.24 ± 0.53
**Nile red**	
Wild type (WT)	2.43 ± 0.13
*Cagpi19* null	1.13 ± 0.04

The average correlation curves were fit to a two-dimensional two-component diffusion model along with a triplet component. The first component of diffusion time for yEmRFP-Ras1 (*τ*_*D1(Ras)*_) was fixed at 125 μs. The first component of diffusion time for Nile red (*τ*_*D1(NR)*_) was fixed at 55 μs. The values shown here are ±standard deviations (S.D).

### Ras1 dynamics in an Hsp90 deficient strain is also slow

**Figure 3:**
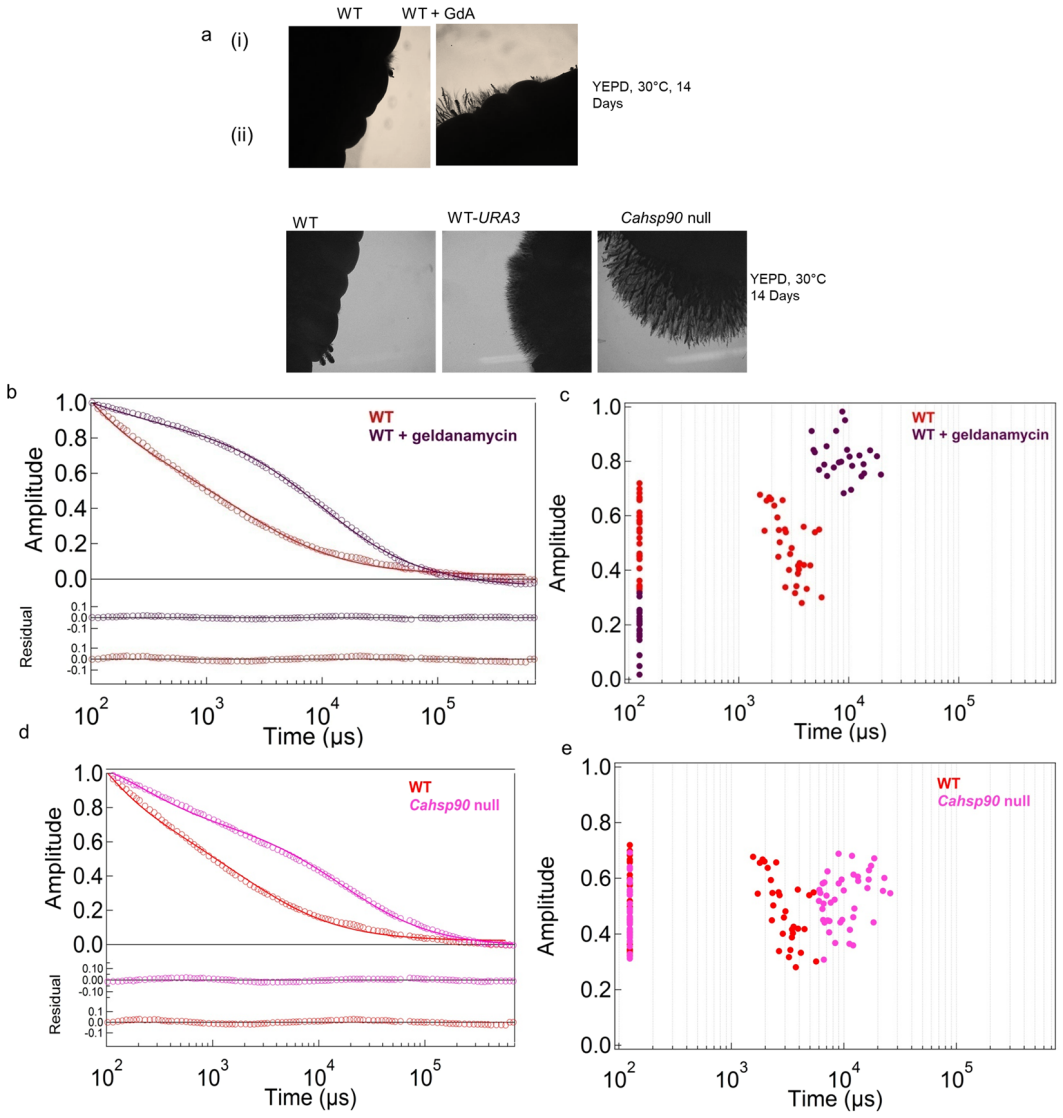
Hyperfilamentous growth and slower Ras1 dynamics in Hsp90 deficient cells. (**a**) Filamentation pattern of (i) wild type (WT) cells treated with the Hsp90 inhibitor geldanamycin (GdA) and of (ii) *Cahsp90* null mutant. The experiment was repeated thrice and representative images are shown. (**b**) Average fluorescence autocorrelation *G*(τ) curves after treatment of wild type (WT) cells with an Hsp90 inhibitor (geldanamycin). (**c**) Plot of amplitude versus diffusion times for the same FCS data obtained for individual cells. (**d**) Average fluorescence autocorrelation G(τ) curves and (**e**) plots of amplitude versus diffusion times in individual cells of *Cahsp90* null mutant. The data was collected for 30 cells in each case.

An alternative method of generating hyperactive Ras signaling is to inhibit the conversion of Ras1(GTP) to Ras1(GDP) and keep it engaged with its effectors. As mentioned earlier the heat shock protein, Hsp90, modulates interaction of Ras1(GTP)-Cyr1 complex with its effectors by inhibiting productive interactions between Ras1(GTP) and Cyr1, thereby promoting its interaction with Ira2 (Fig. 1b)^11^. The amount of active Ras1 available for interacting with Cyr1 is thus a function of the levels of Hsp90 in the cell. *C. albicans* mutants defective in Hsp90 are inefficient in turning off the activated form of Ras1 and producing hyperfilamentation, a hyperactivate Ras1 phenotype^10^. Hsp90 levels in the fungal cell can also be altered chemically by incubating the cells with the Hsp90 specific inhibitor, geldanamycin^18^. For the purpose of this study, both approaches were used. Treatment of the wild type strain with geldanamycin caused the cells to exhibit hyperfilamentation (Fig. 3a(i)). Generating a conditional null *Cahsp90* mutant also gave a hyperfilamentous phenotype (Fig. 3a(ii)). Diffusion dynamics of Ras1 in these strains also gave fluorescence auto-correlation curves that could be fit to a 2D2C diffusion model (equation 5). In both cases, membrane-bound Ras1 exhibited significantly slower diffusion times, *τ*_*D2*_ (Fig. 3b–e; Table 1), which closely resembled that of Ras1G13V.

### Ras Hyperactivation involves actin polymerization

**Figure 4:**
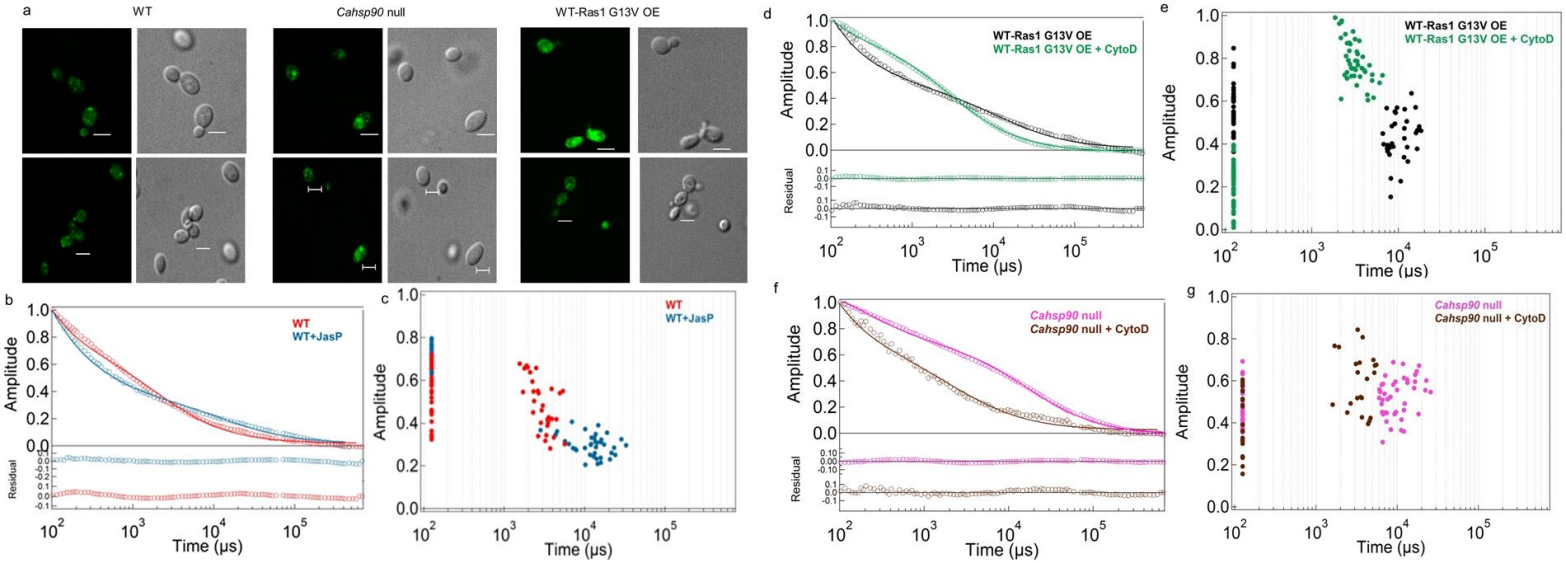
Altered actin polymerization upon Ras1 hyperactivation might cause slower Ras1 dynamics. (**a**) Microscopic images showing the staining of total actin in the wild type, *Cahsp90* null and the WT-Ras1G13V OE strains. A β-actin primary antibody and secondary goat anti-mouse FITC labelled antibody was used to visualize the total actin of the cell. Scale bar corresponds to a distance of 5 µm. (**b**) Average fluorescence autocorrelation *G*(τ)curves after treatment of wild type (WT) cells with an inducer of actin polymerization, jasplakinolide (JasP). (**c**) Plot of amplitude versus diffusion times for the same FCS data obtained for individual cells. (**d**,**e**) Average fluorescence autocorrelation curves *G*(τ) and plot of amplitude versus diffusion time in individual cells of overexpressing Ras1G13V, before and after treatment with Cyto D. (**f**,**g**) Fluorescence autocorrelation curves *G*(τ) and a plot of amplitude versus diffusion time of *Cahsp90* null strain before and after treatment with Cyto D. The data was collected for 30–40 cells in each case.

In *S. cerevisiae*, actin polymerization is known to cause Ras hyperactivation^19^. Could Ras hyperactivation also induce actin polymerization? Actin in the strains with hyperactive Ras phenotypes were visualised using anti-β-actin antibodies. Actin was polymerised to a greater extent in the strain overexpressing Ras1G13V as well as in the *Cahsp90* null as compared to the wild type (Fig. 4a and Supplementary Fig. 4). Cytochalasin D (Cyto D), an inhibitor of actin polymerization, is known to promote depolymerisation of actin^20^ while jasplakinolide (JasP) promotes actin polymerization^21^. Hence, wild type strain was treated with JasP while the strain overexpressing Ras1G13V was treated with Cyto D and their fluorescence auto-correlation curves recorded. The data were analysed as before (Fig. 4b–e). Significantly slower dynamics (*τ*_*D2*_) was observed in the wild type strain treated with JasP while faster dynamics was observed in the strain overexpressing Ras1G13V upon treatment with Cyto D (Table 1). Similar results were obtained in the *Cahsp90* null treated with Cyto D as well (Fig. 4f,g; Table 1). All these results suggest that hyperactivation of Ras1 via depletion of Hsp90 involves actin polymerization.

### Dynamics of Ras1 in a hyperfilamentous sterol-deficient strain of *C. albicans* suggests hyperactivation of Ras1

Next we examined a rather unusual strain of *C. albicans*. This strain was generated by downregulation of *CaGPI19*, a gene encoding one of the subunits of the multi-subunit enzyme complex (GPI-*N*-acetyl glucosaminyltransferase) that catalyzes the first step of GPI biosynthesis in *C. albicans*. Heterozygous and conditional null *Cagpi19* mutants show hyperfilamentation that could be specifically correlated with higher Ras- dependent cAMP/PKA signaling^22,23^.

**Figure 5:**
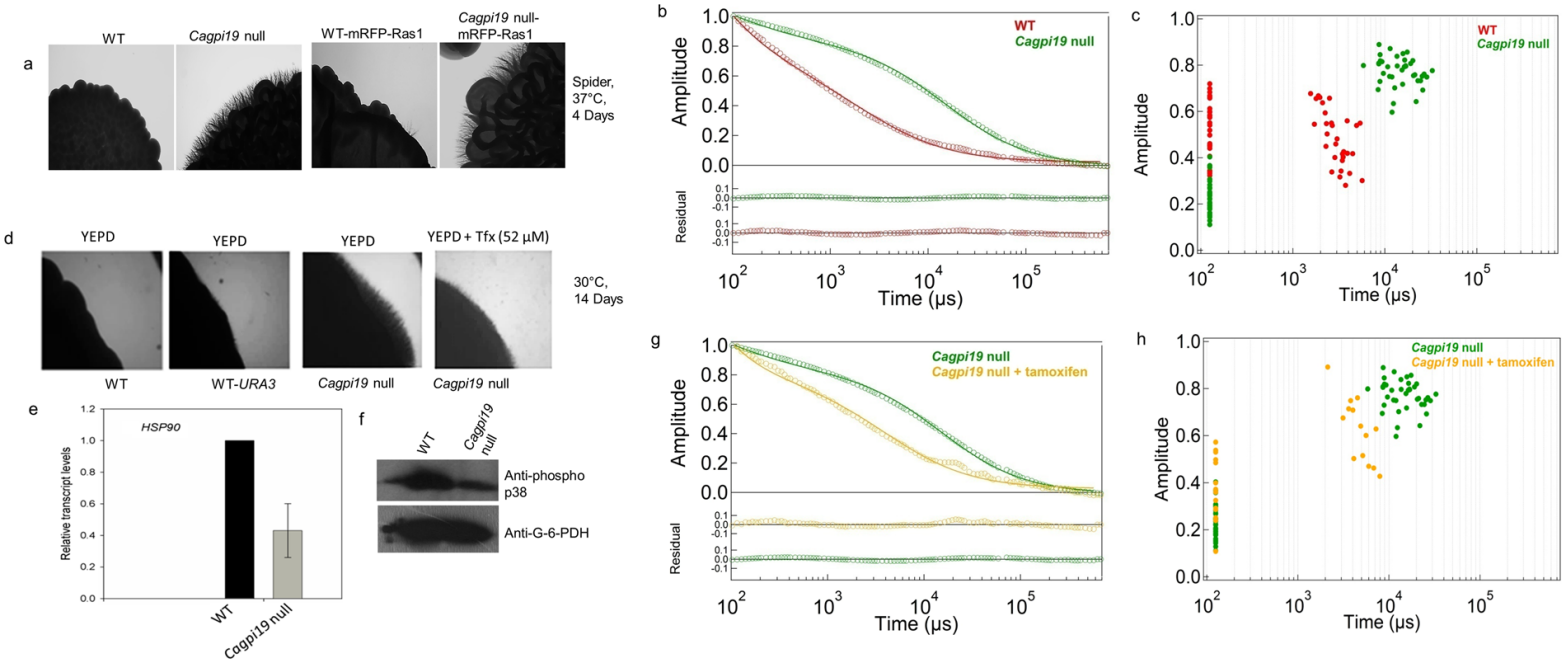
Hsp90 is also responsible for slower Ras1 dynamics in the *Cagpi19* null mutant. (**a**) Phenotypic studies showing that tagging Ras1 with mRFP at the N-terminus does not alter its functionality and does not alter the hyperfilamentous phenotype of the mutant strain at 37 °C. (**b**,**c**) Fluorescence autocorrelation curves *G*(τ) and a plot of amplitude versus diffusion times after treatment of wild type (WT) and *Cagpi19* null strains. The data were collected for 35–40 cells. (**d**) Hyperfilamentation phenotype of the *Cagpi19* null mutant at 30 °C relative to WT strain is reversed upon addition of tamoxifen (TfX), an Hsp90 activator. The experiment was done thrice and representative images are shown. (**e**) Quantification of the transcript levels of *CaHSP90* in the *Cagpi19* null strain by quantitative PCR method. The experiment was done thrice in duplicates and average values along with standard deviations are shown. (**f**) Western blot showing a decrease in the levels of phosphorylated Hog1 in the *Cagpi19* null relative to the wild type. G-6-PDH was taken as the loading control. The experiment was done thrice and a representative blot is shown. The complete images of the two western blots are shown in Supplementary Fig. 5. (**g**,**h**) Average fluorescence autocorrelation G(τ) curves and plots of amplitude versus diffusion times in individual cells after treating the *Cagpi19* null with tamoxifen. The data was collected for about 15 cells after addition of tamoxifen.

Ras1 in *Cagpi19* null was tagged with mRFP and was seen to localize to the plasma membrane (Supplementary Fig. 1a). The strain continued to be hyperfilamentous as compared to control strains (Fig. 5a). The diffusion dynamics of Ras1 in yeast cells of the mutant strain vis-à-vis the wild type (WT) strain was monitored by FCS. Ras1 had considerably slower diffusional dynamics (*τ*_*D2*_) in *Cagpi19* null as compared to that in wild type (WT) cells (Fig. 5b,c; Table 1). Interestingly, the diffusion times (*τ*_*D2*_) were comparable to those observed for Ras1G13V as well as those for Ras1 in conditions of Hsp90 deficiency (see Table 1). This would suggest that Ras1 in *Cagpi19* null is also constitutively activated.

### Ras1 hyperactivation in *Cagpi19* conditional null mutant involves actin polymerization

How is Ras hyperactivation achieved in the *Cagpi19* null? A closer examination of the phenotypes of this mutant would suggest that it phenocopies *hsp90* mutants in *C. albicans* (Fig. 5d). Like the *hsp90* deficient mutants^10^, *Cagpi19* cells show filamentation at 30 °C in strong hyphae inducing medium like Spider (data not shown). Milder inducing agents like glucose in YEPD can also induce hyphal growth at 30 °C if the cells are incubated in them for longer times (over a week or more; Fig. 5d). Their cell walls show chitin accumulation and they are heat shock sensitive^23,24^. These phenotypes suggest a downregulation/inactivation of *HSP90* in *Cagpi19* null. Indeed, we found a reduction in the transcript levels of *HSP90* in the *Cagpi19* null (Fig. 5e). To assess the activity of Hsp90, we probed the levels of phosphorylated Hog1, a well-known Hsp90 client protein in *C. albicans* using a phospho-p38 MAPK (Thr180/Tyr182) antibody^25,26^. The levels of phosphorylated Hog1 were lower in *Cagpi19* null (Fig. 5f). Moreover, when cells of *Cagpi19* null were treated with tamoxifen, an activator of Hsp90^27^, the hyperfilamentous phenotype of *Cagpi19* null mutant could be reversed (Fig. 5d). It also resulted in faster diffusion of mRFP-Ras1 in *Cagpi19* null cells (Fig. 5g,h). Thus, in *Cagpi19* null the levels of Hsp90 appear to be reduced, leading to hyperactivation of Ras1.

**Figure 6:**
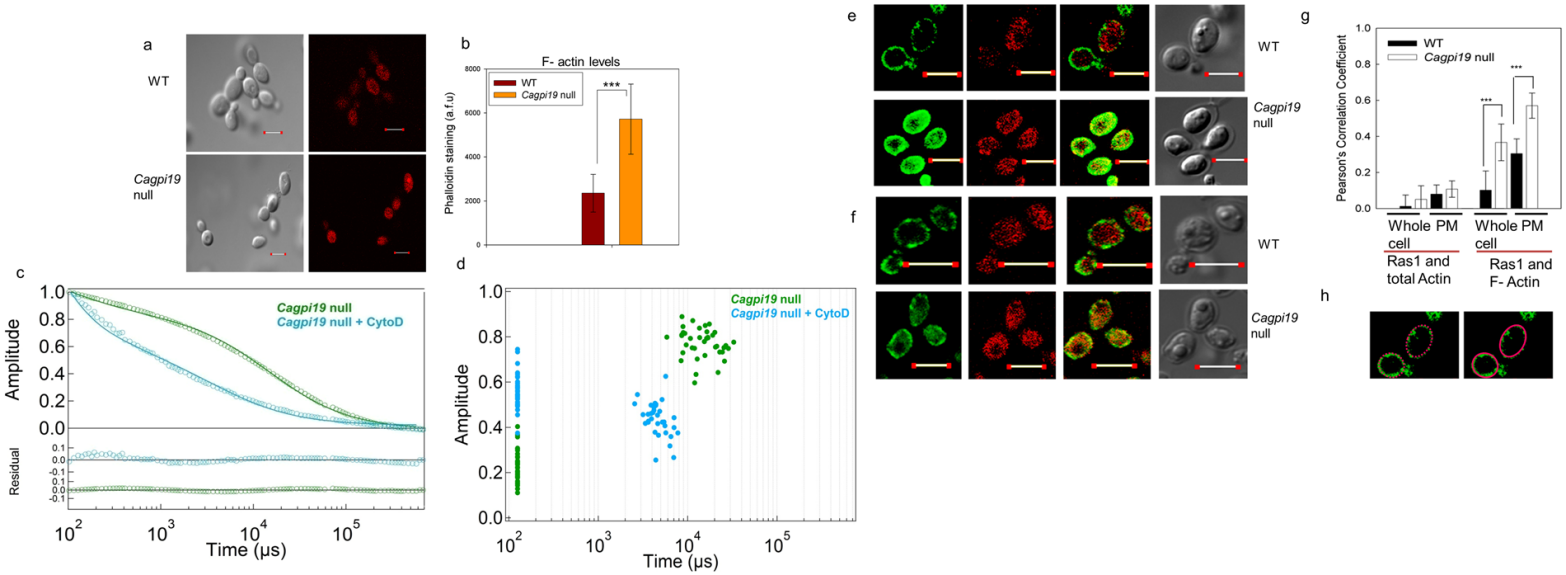
Increased actin polymerization in *Cagpi19* null causes slower Ras1 dynamics. (**a**) Immunostaining of polymerized actin filaments using rhodamine-phalloidin in the wild type (WT) and the *Cagpi19* null mutant. Scale bar corresponds to a distance of 5 µm. (**b**) Quantification of the F-actin levels in the *Cagpi19* null relative to the wild type estimated through rhodamine-phalloidin staining. In each case 40 cells were taken for quantification. Students’ t-test was used to calculate the p value (***p value = 1.27 × 10^−9^). (**c**,**d**) Average fluorescence autocorrelation curves G(τ) and plot of amplitude versus diffusion times in individual cells after treating the *Cagpi19* null with Cyto D. The data was collected for more than 25 cells in each case. (**e**) Colocalization of Ras1 with the polymerized F-actin in the *Cagpi19* null and the wild type using anti-Ras1 and rhodamine-phalloidin. (**f**) Colocalization of Ras1 with the total β-actin in the *Cagpi19* null and the wild type using anti-Ras1 and anti β-actin antibody. Scale bar corresponds to a distance of 5 µm. (**g**) Quantification of the extent of colocalization of Ras1 with F-actin/total actin using the Pearson’s correlation coefficient. A minimum of 40 cells were taken for quantification in each case. PM indicates plasma membrane. Scale bar corresponds to a distance of 5 µm. Students’ t-test was used to calculate the p value(***p value = 2.25 × 10^−7^ for PM; ***p value 3.98 × 10^−7^ for whole cell). (**h**) Figure showing a representation of the selection of ROI (region of interest) for calculating the Pearson’s correlation coefficient. The left panel shows the selection for calculating the correlation for the plasma membrane. Points all over the plasma membrane were selected for analysis. The right panel shows the selection of the area for calculating the correlation for the whole cell. ROI was selected such that the whole cell was considered for the analysis. Pearson’s correlation coefficient was then calculated using Fluo View software.

Does Ras1 hyperactivation in *Cagpi19* null also involve actin dynamics? To examine the polymerization status of actin we stained the cells with rhodamine-tagged phalloidin. The *Cagpi19* null showed nearly two-fold higher phalloidin staining as compared to wild type (Fig. 6a,b), clearly indicating the presence of higher levels of F-actin in these cells. To confirm that the alteration in dynamics was due to the altered polymerization status of cellular actin, the *Cagpi19* null strain was treated with Cyto D. Significantly faster Ras1 dynamics was observed upon treatment with Cyto D (Fig. 6c,d; Table 1). Similar results were observed when *Cagpi19* null was treated with latrunculin B (Lat B), another inhibitor of actin polymerization^28^ (Supplementary Fig. 6; Table I). Thus, altering actin polymerization status can alter Ras1 dynamics.

Previous reports in live cells suggest that actin polymerization causes a change in viscosity^29^. This could also affect the dynamics of CaRas1, if viscosity of the cytoplasm changes upon actin polymerization. Does viscosity change upon actin polymerization in *C. albicans* cells? To test this hypothesis, we assessed the alteration in rotational dynamics of a cell-permeable dye, calcein-AM, using fluorescence anisotropy. We observed a roughly 2-fold increase in steady-state anisotropy for the dye (r = 0.26 in the *Cagpi19* null, r = 0.11 in the wild type), suggesting that viscosity increase could contribute to some extent to the slower dynamics of CaRas1.

### Ras1 co-localizes with G-actin rather than F-actin

Phalloidin staining in the *Cagpi19* null strain showed significant amounts of F-actin in *Cagpi19* null whereas the wild type strain was poorly stained (Fig. 6a,b). Yet, an analysis of Pearson’s correlation coefficients for Ras1 and F-actin suggests poor co-localization of Ras1 with F-actin in *Cagpi19* null (Fig. 6e). Ras1 did, however, show significant co-localization with β-actin at the plasma membrane of *Cagpi19* cells relative to wild type cells when anti-β-actin antibodies (which does not distinguish between monomeric and polymerized forms of actin) were used for the detection (Fig. 6f–h). Thus, we infer that hyperactivated Ras1 preferentially co-localizes with G-actin rather than F-actin.

### Ergosterol plays no role in Ras1 dynamics

Membrane sterols influence dynamics of certain Ras isoforms in mammals. For example, the inactive form of H-ras (GDP-bound) associates with cholesterol-dependent domains in the inner membrane while its active form (GTP-bound) does not^30,31^. K-ras on the other hand shows no cholesterol-dependent association with microdomains in the plasma membrane^32^. Do membrane sterol levels influence Ras1 dynamics in *C. albicans*? This question is particularly important in the case of the *Cagpi19* null strain which is ergosterol-deficient due to downregulation of *ERG11*, a key enzyme required for ergosterol biosynthesis^22^.

Despite ergosterol deficiency, this strain has slower Ras1 dynamics as shown above. One straight forward inference from these results would be that ergosterol does not influence Ras1 dynamics. However, it is also possible that the sterol depletion in this strain is insufficient for an alteration in Ras1 dynamics. GC-MS analysis suggested that ergosterol levels in *Cagpi19* null were ~50% lesser as compared to the wild type (Supplementary Fig. 7a). Was this extent of sterol depletion significant for alteration in membrane packing/dynamics as reported for ergosterol mutants^33,34^?

To study whether membrane organization had been perturbed in the mutant strain, the lateral diffusion of Nile red, a membrane-specific probe, within the plasma membrane of the wild type (WT) and *Cagpi19* null was studied using FCS (Supplementary Fig. 7b,c). For Nile red also two well resolved diffusion times were observed when fitted with a 2D2C diffusion model, incorporating a triplet component of 4 μs (equation 5). Of these, the component with the faster diffusion time was fixed at 55 μs which corresponds to the diffusion of free Nile red in buffer. The second diffusion time-component *τ*_*D2(NR)*_) for Nile red in the *Cagpi19* null strain was nearly two-fold lower on average than that for the probe in the membrane of wild type (WT) cells (Table 1). This suggests that the membrane of the conditional null *Cagpi19* mutant is poorly packed and permits faster diffusion of Nile red.

These results were further confirmed using steady state fluorescence anisotropy and lifetime studies with diphenylhexatriene (DPH), another small molecular weight hydrophobic probe used as a reporter of the global state of fluidity and packing of membrane^35^. The lifetime of DPH is extremely sensitive to the polarity of the microenvironment it experiences and it is known to be shortened in solvents with high dielectric constants, such as water. The probe displayed multiexponential decays in the cell membranes, which is also reported in literature previously^36^. The data could be fitted well to tri-exponential decays, as determined by reduced chi-square (*χ*^2^) values (Supplementary Fig. 8). The long lifetime is reflective of an environment with lower dielectric constant and higher viscosity, and we interpret this to come from the fraction of fluorophore embedded deep inside the membrane core. The medium lifetime component may reflect a fraction of the probe that is not as deeply buried and the short lifetime component, due to a polar environment, may represent the fraction of the fluorophore close to the membrane-water interface. Upon comparison of the lifetimes of DPH in the membrane of the conditional null, there was an decrease in *τ*_*1*_ and *τ*_*2*_ (0.310 ns and 2.12 ns in the wild type as compared to 0.167 ns and 1.48 ns, respectively, in the *Cagpi19* null) while the fractions contributing to each of them, *f*_1_ and *f*_2_, increased (26% and 14% in the wild type increased to 55% and 26%, respectively, in *Cagpi19* null). On the other hand, as can be also seen from Supplementary Table I, the contribution of the longest lifetime (*τ*_3_) was much smaller in the *Cagpi19* null (*f*_3_~ 19%) as compared to the wild type (*f*_3_~ 60%). Overall, the average lifetime was also much shorter in the *Cagpi*1*9 null* (1.99 ns) as compared to the wild type (5.11 ns). The most plausible explanation for these results is that the decrease in ergosterol levels leads to poor membrane packing, causing greater water penetration in the cell membranes of the mutants. Being hydrophobic, DPH is known to prefer locations close to the acyl chains with its axis aligned parallel to them^37^. Greater water penetration in the cell membrane results in environments with higher dielectric constants for a greater fraction of DPH molecules in the membrane of *Cagpi19* null. Unlike in FCS where we monitor translational diffusion of a few (2–5) probe molecule(s) over relatively long distances, in fluorescence lifetimes and steady state anisotropy measurements, the data obtained are ensemble averages and the probes report on their immediate microenvironment rather than on long-range bulk membrane properties provided by the random walk of fluorescent probes. Thus, the membrane of *Cagpi19* null appears considerably perturbed with reference to short- and long-range order. Yet, as mentioned earlier, Ras1 in this strain exhibited slower dynamics.

Is Ras1 dynamics influenced by ergosterol? In order to probe this, WT cells were depleted of ergosterol by treatment with β-cyclodextrin (βCD) (confirmed by GC-MS; Supplementary Fig. 7a) and Ras1 dynamics was examined by FCS. No significant variation in Ras1 dynamics was observed (Supplementary Fig. 9; Table I) despite ~50% ergosterol depletion. This would suggest that ergosterol *per se* is unlikely to influence the mobility of Ras1 in *C. albicans*.

## Discussion

As explained in the Introduction, Ras is a small GTPase, highly conserved from lower eukaryotes to humans. It cycles between an inactive GDP-bound and an active GTP-bound state triggering multiple signaling events. Defects in Ras signaling can lead to many developmental disorders and cancers. While it works via many downstream pathways in *C. albicans* as well, the major focus in this organism has been the cAMP mediated PKA activation pathway due to its importance in the yeast to hyphal transition in response to various stimuli. Of particular relevance to this study are situations in which Ras is overactive or hyperactivated. Based on the results presented above we argue that not all modes of Ras activation have identical consequences.

Using FCS, we demonstrate that overexpression of *C. albicans* Ras1 is dynamically a very different event from Ras1 hyperactivation that involves either constitutive activation of Ras1 or inhibition of downstream steps that promote the interaction of active Ras1 with GAPs. Endogenous as well as overexpressed Ras1 follow very similar dynamics in the plasma membranes which is substantially faster than the dynamics of constitutively activated Ras1G13V in *C. albicans*. In the former case, Ras1 exhibits diffusion times of 2–4 ms. In the latter case, Ras1 exhibits diffusion times of 12–17 ms. This is also the case in an Hsp90 deficient strain of *C. albicans*. Geldanamycin, an inhibitor of Hsp90, can also produce slower Ras1 diffusion in wild type *Candida* cells.

How is this change in dynamics of Ras1 effected? In *S. cerevisiae*, actin polymerization results in hyperactive Ras signaling^19^. We wondered whether the converse may be true in *C. albicans*. We observed higher actin polymerization in cells with hyperactivated Ras1. Treatment with Cyto D, an actin depolymerization agent significantly shortened the *τ*_*D*2_ values for Ras1, making its dynamics resemble that of Ras1 in wild type cells. Likewise, treatment of wild type cells with jasplakinolide, an actin polymerization agent, results in slowing down of Ras1 dynamics. Thus, we see a correlation between actin polymerization and slower Ras1 dynamics. A very similar result was observed in the hyperfilamentous GPI mutant, *Cagpi19* null. Our data suggests that this mutant is deficient in Hsp90 and has higher levels of polymerized actin. As expected, the dynamics of Ras1 is significantly slower in these cells. When *Cagpi19* null cells were treated with tamoxifen, an activator of Hsp90, the diffusion times were comparable to that seen in wild type cells. Thus, Ras1 hyperactivation and actin polymerization seem to be interlinked.

Actin polymerization also increases the viscosity of the cytoplasm by roughly two-fold. The extent of viscosity changes seem to be roughly similar to what has been reported in literature. For example, in Dictyostelium, a 2-fold increase in diffusion of soluble GFP and a 1.5-fold reduction in cytoplasmic viscosity was observed in cells treated with an actin polymerization inhibitor^29^. This increase in viscosity alone cannot account for the 4- to 6-fold altered dynamics of Ras1 that we observe. Analysis of Pearson’s coefficients in co-localization studies suggest that Ras1 does not co-localize with F-actin and instead seems to co-localize with G-actin.

It is well known that the cAMP/PKA pathway can be turned on by Ras1 via its effector Cyr1 and Cap1 (cyclase associated protein 1). This binding is GTP-dependent and independent of post-translational modifications of Ras1^38^. The cAMP/PKA signaling can also be turned on in a Ras1-independent manner by Cyr1 involving Cap1 and G-actin while inducing F-actin polymerization^39^. Literature reports suggest that both Ras1-Cyr1-Cap1 and Cyr1-Cap1-G-actin complexes are stable enough to be isolated^38,40^. However, no one has been able to isolate a Ras1-Cyr1-Cap1-G-actin complex so far^39,40^. We too were unsuccessful in isolating such a complex. It is possible that these interactions are transient and occur only during the signaling process. Disrupting the cell for co-immunoprecipitation probably abrogate these interactions. Nevertheless, based on the co-localization studies and the FCS data, we conclude that actin polymerization is a consequence of Ras1 hyperactivation and *vice versa*. In other words, the two paths may be coupled if either Ras1 is hyperactivated or when actin polymerization is triggered, causing the cAMP/PKA pathway to stay turned on. This may be achieved in strains expressing Ras1G13V or where Hsp90 is inhibited/downregulated by the recruitment of G-actin to the Ras1-Cyr1-Cap1 complex. Alternatively, this may also be achieved by activating actin polymerization (as we show using JasP) wherein Ras1 may be recruited to the Cyr1-Cap1-G-actin complex. Thus, formation of this complex provides the mechanism by which cAMP/PKA signaling pathway stays turned on for longer periods, long enough for hyphal growth to be established even in the absence of strong hyphal inducing cues. The slowing down of Ras1, resulting in *τ*_*D2*_ of 12–17 ms, is also due to the formation of this larger complex. Thus, both path I and path II of Fig. 1b may be coupled in these cells.

Recent work from the Cowen lab provides support for such a hypothesis wherein they observed synergy in the action of Hsp90 inhibitors and actin inhibitors in fungal cells^26^. In addition, they showed that Hsp90 downregulation affects localization of Wal1, an actin nucleation promotion factor, which affects actin filamentation dynamics^26^. Interestingly, in actin-stabilized *S. cerevisiae* cells, hyperactivation of Ras signaling was observed which depended on the ability of its Cap (Srv2) to bind G-actin at its C-terminus^19^. Polarized actin, typically required for hyphal growth, was also observed in *S. cerevisiae* cells treated with the Hsp90 inhibitor, geldanamycin^41^.

On the other hand, in the presence of Hsp90/Sgt1 the interaction between Ras1(GTP) and Cyr1 is inhibited and Ras1 switches back rapidly to its OFF state. It is this species that we detect as having diffusion times of 2–4 ms in the cytoplasm of wild type cells or cells overexpressing wild type Ras1. Since downstream actors are not engaged for long enough, only basal Ras signaling occurs in these cells. This could also explain why actin appears well dispersed in the cytoplasm and why these cells do not spontaneously produce hyphae at ambient temperature.

That the hyperactivated form of Ras1 in *C. albicans* is a slower diffusing species than wild type Ras1 (whether overexpressed or not), is contradictory to what the Hogan group reported^16^. These differences perhaps arise due to differences in the experimental design, the sensitivities of the techniques used and the time scales for which Ras1 dynamics is tracked in their experiments versus ours. Prof. Hogan’s team focused on fluorescence recovery after photobleaching (FRAP) at polarized bud tips in yeast cells, which are also sites for localization of specialized proteins that guide bud growth^42,43^. Ras1 molecules present at these sites are probably specifically interacting with the Cyr1-Cap1-G-actin complex to polymerize actin at the cortical tips, a hypothesis that the authors too have proposed^16^. On the other hand, we did not focus on bud sites. Ras1 and Ras1G13V would have very similar dynamics, if this were the case.

The dynamics of Ras1 in the *Cagpi19* null, a hyperfilamentous GPI anchor biosynthetic mutant, can also now be rationalized. Recent reports suggest that a *bona fide* interactor of Hsp90 is *ERG11*, a crucial gene in the sterol biosynthetic pathway^26^. We have previously shown that *CaGPI19* and *ERG11* are mutually co-regulated^22^. Thus, the hyperactive Ras phenotypes of *Cagpi19* null are probably mediated via the interaction between *ERG11* and *HSP90*. As expected in an *ERG11* deficient strain, the *Cagpi19* null is ergosterol deficient. Nevertheless, the dynamics of hyperactivated Ras1 in this strain are comparable to that of Ras1 in the *hsp90* null or Ras1G13V in wild type cells. This indicates that ergosterol does not affect the dynamics of hyperactivated Ras1. Recent reports show that supplementing sterol-deficient *erg* mutants with ergosterol does not alter their Hsp90 deficient status either^26^. Treating wild type cells with βCD, as done by us, to sequester the ergosterol from the membrane also caused no change in Ras1 dynamics, indicating that ergosterol *per se* does not influence Ras dynamics.

Differentiating between overexpressed and hyperactivated Ras could have major implications for physiological signaling processes, not only in fungal systems but also in other eukaryotic systems, including humans. The time scales of FCS measurements permit us to observe the dynamics of such events reliably. Understanding the dynamics that underlie these processes should also provide better insights into the process of Ras signaling and assist in the development of new rapid diagnostics as well as in the design of Ras inhibitors.

## Materials and Methods

### Materials

Chemicals used were of analytical grade and procured from Sigma Aldrich, Fluka, Merck or SRL. Growth media were purchased from Himedia and Qualigens. The primers used for the study were synthesized by Sigma-Aldrich/GCC Biotech.

### Strains and media

The control BWP17 strain of *C.albicans* was a kind gift from Prof. Aaron Mitchell^44^. The heterozygote (*Cagpi19/CaGPI19*) and conditional null (*Cagpi19/MET3-GFP-CaGPI19*) strains were derived from *C. albicans* BWP17 (wild type (WT)) and grown in minimal medium containing 5–10 mM Met/Cys to repress the expression of *CaGPI19* as described previously^22,24^. The heterozygous *ERG11* mutant (*erg11/ERG11*), in BWP17 strain, where one allele of the gene was knocked out has been described previously^22^. *Cahsp90* conditional null was generated by disrupting one allele of *CaHSP90* with *HIS1* marker using primers CaHSP90 FP and CaHSP90 RP and placing the second allele under the control of regulatable *MET3* promoter using the *pMET3-URA3-GFP* cassette^45^ using primers CaHSP90 NULL FP and CaHSP90 NULL RP. The strain was then grown in minimal medium containing 5–10 mM Met/Cys to repress the expression of *CaHSP90*.

### Generating mRFP-Ras1 in *C. albicans* strains

The plasmid mRFP-ARG4-mRFP was constructed by cloning the two halves of yEmRFP^46^ (from CIp10 ADH1-Cherry plasmid, a kind gift from Prof. Neta Dean, Stony Brook University) on either side of the ARG4 selection marker using RFPFF FP, RFPFFRP, RFPSF FP and RFPSF RP. This cassette was then amplified using Ras1-RFP-FP and Ras1-RFP-RP (Supplementary Table II) and transformed into *C. albicans* BWP17 and the conditional null *Cagpi19* mutant (*Cagpi19/MET3-GFP-CaGPI19*) such that the cassette integrated downstream of the native Ras1 promoter. The colonies obtained were grown for four generations on synthetic medium supplemented with arginine, so that the *ARG4* could be removed after reconstitution of yEmRFP. The colonies were screened by fluorescence to check for the expression of yEmRFP-Ras1 The same CIp10 ADH1-Cherry plasmid was digested with StuI and transformed in *C. albicans* to generate the cytosolic mRFP expressing strain.

### Overexpression strains

Ras1 overexpression strains were created by inserting the pADH1-RFP-Ras1/pADH1-RFP-Ras1G13V plasmid at the *RPS1* locus of *C.albicans*. pADH1-RFP-Ras1 plasmid was constructed by amplifying Ras1 from pEA-GFP-Ras1^16^ with primers Ras1 FP and Ras1 RP and cloning between HindIII and NheI in pEGFP to give rise to pADH1-Ras1. mRFP was amplified from pCIP10-ADH1-Cherry and cloned in PstI site of pADH1-Ras1 using RFP-FP and RFP-RP. The plasmid pADH1-RFP-Ras1G13V was then made by site-directed mutagenesis using DpnI method^47^ with primers Ras1G13V FP and Ras1G13V RP. The primer sequences are given in Supplementary Table II. The plasmids were digested with StuI to linearize them and then transformed in *C. albicans*.

### Filamentation assay

Cells were grown to log phase in SD minimal medium and cells corresponding to 0.2 OD_600nm_ were spotted on Spider/YEPD/indicated plates and incubated at appropriate temperatures and images were taken at the indicated time in Nikon SMZ1500 microscope.

### Microscopy

For visualizing mRFP, cells were grown to log phase in SD minimal medium, spotted on slides and images were captured in Nikon 90i/TiE microscope/Nikon 1X71 confocal microscope

### DPH labeling of strains

Strains were grown to late-log phase, washed with water and spheroplasted in lyticase-buffer (10 units/ml of lyticase in 1 M Sorbitol, 65 mM KH_2_PO_4_) at 25 °C for an hour. After washing, cells were resuspended to an OD_600 nm_ of 1 in 10 mM PBS containing 2 μM DPH (Sigma Aldrich). Cells were stained in the dark for 20 minutes with gentle shaking, then washed and resuspended to an OD_600 nm_ of 0.4–0.6 for fluorescence lifetime and anisotropy measurements. For microscopic imaging, the same cells were spotted on microscopic slides and visualized under Nikon 90i/TiE microscope/Nikon 1X71 confocal microscope. When required, the cells were incubated with 20 mM βCD for 1 h before staining with DPH.

### Treatment of cells with methyl β-cyclodextrin (βCD) for membrane dynamics

Spheroplasted cells as described above, were treated with 17.6 mM βCD in buffer for 1 h at room temperature. The depletion of ergosterol levels was confirmed by GC-MS.

### Steady state anisotropy measurements

Anisotropy of DPH was measured on a Cary Varian Eclipse spectrofluorimeter with a manual polarizer attachment. Steady state anisotropy, *r*_*ss*_, was measured as

1 $${r}_{SS=\frac{{I}_{VV}-G{I}_{VH}}{{I}_{VV}+2G{I}_{VH}}}$$

where, *I* is the fluorescence intensity measured with the excitation polarizer in vertical (*V*) position and the emission polarizer in either vertical (*V*) or horizontal (*H*) position. *G* is the grating factor used to account for the detector sensitivity to vertical and horizontal polarized light. *G* was measured as described^35^ and is given by

2 $$G={I}_{HV}/{I}_{HH}$$

### Time-resolved fluorescence measurements

Fluorescence lifetimes were recorded on an Edinburgh Instruments FL920 with a TCSPC card (TCC-900) and a picosecond diodelaser (375 nm, pulse-width 60 ps; EPL-375, Edinburgh Instruments). The instrument response function (IRF) was measured using a solution of Ludox^®^ and was ~85 ps. Lifetime decays were obtained by setting the emission polarizer to 54.7° from the vertical (magic angle) to avoid artifacts caused by rotational anisotropy. DPH was excited at 358 nm and its emission was detected at 430 nm. The raw decay data were analyzed using the equation:

3 $$I(t)={f}_{1}\exp (-\frac{t}{{\tau }_{1}})+{f}_{2}\exp (-\frac{t}{{\tau }_{2}})+{f}_{3}\exp (-\frac{t}{{\tau }_{3}})$$

where, *f*_*i*_ are the normalized pre-exponential factors representing the fractional contribution of the lifetime components *τ*_*i*_. Lifetime components were extracted by deconvoluting the IRF in a least-square fitting program based on the Marquardt algorithm. The reduced chi-square (*χ*^2^) values were significantly improved when a three-exponential fit was used compared to either mono- or bi-exponential decays. The number-averaged mean lifetimes were calculated using:

4 $$\langle \tau \rangle =\sum _{i}{f}_{i}{\tau }_{i}$$

### Fluorescence correlation spectroscopy measurements

A home-built fluorescence correlation spectrometer (FCS) set-up based on Olympus IX71 inverted fluorescence microscope with a 60X water-immersion objective (NA 1.2, UPlanSApo, Olympus) was used^48^. Instrument calibration- The FCS instrument was calibrated using Rhodamine-6G (R6G) in water. R6G was excited using 532 nm DPSS laser (CNI Optoelectronics Tech. Co., Ltd.). The fluorescence bursts from the dye were passed through a dichroic (Model-XF2016, Omega Optical Inc., USA) and emission filter (Model-607AF75, Omega Optical Inc., USA) to block the excitation light and fed into a multi-mode fiber patch cord (Model-QMMJ-3S3S-UVVIS-25/125-3-1, Oz Optics, Canada) of diameter 25 µm, which acts as the confocal pinhole. The fluorescence signal was detected using a single photon avalanche photo diode (Perkin Elmer, SPCM-AQRH-13-FC). The autocorrelation of the fluorescence bursts were obtained by using FLEX correlator card (FLEX990EM-12D, Correlator.com, USA) and collected in a routine written in LabView. The correlation curves for R6G were fitted with 3-D diffusion model^48–51^ to extract its translational diffusion time (*τ*_*D*_) through the observation volume, which gave a value of *τ*_*D*_ = 63 µs. From this value and the known diffusion constant of R6G, the lateral resolution of the setup was determined to be ~285 nm and the observation volume was calculated to be ~1.01 fL^48,52^.

### Nile Red dynamics

Overnight grown late-log phase cells were incubated with 0.4 μM stock solution of a membrane-selective dye, Nile Red (Sigma Aldrich), in water for 45 minutes and washed twice prior to placing them on a poly-lysine-coated cover slip and sealing them on a glass slide. The laser (532 nm) was focused specifically in the membrane regions of the cells to excite the membrane-bound Nile Red. Moving the laser focus out of the cell resulted in either no fluorescence or very low fluorescence whose correlations were very fast, comparable to Nile Red in bulk buffer (*τ*_*D(NR)*_ = ~55µs). The resultant diffusion coefficient was calculated to be 4.5 × 10^−6^ cm^2^/sec and was comparable to that reported previously, viz. 2.9 × 10^−6^ cm^2^/sec^53^. Autocorrelation curves of Nile Red inside the plasma membranes were collected for as many as 10–12 different cells for a particular strain.

### mRFP-Ras1 and Nile Red dynamics

Overnight grown late-log phase yeast cells of wild type (WT) strain and the conditional null mutant of *Cagpi19 *expressing mRFP-Ras1, were taken and given lyticase-buffer treatment for 1 h at 25 °C. The cells were then washed and placed on polylysine coated coverslip and sealed on a glass slide. FCS measurements were carried out for 20–40 cells in a similar fashion as also for the Nile Red stained cells and the correlation curves were fit to the same model (equation 5). When required, the spheroplasted cells were incubated with geldanamycin (10 µM), tamoxifen (52 µM), jasplakinolide (12 µM), latrunculin B (12.5 uM) for 1 h or cytochalasin D (20 µM) for 16 h, washed and then samples were prepared for FCS measurements. The measured correlation curves of mRFP-Ras1 and Nile Red inside membrane displayed the characteristics of typical two-dimensional diffusion^36,54^ and could be fitted well to two-component diffusion model (2D2C), multiplied with a triplet (flickering) contribution as given by

5 $$G(\tau )=\{{g}_{01}{(1+\frac{\tau }{{\tau }_{D1}})}^{-1}+{g}_{02}{(1+\frac{\tau }{{\tau }_{D2}})}^{-1}\}\{1+{A}_{T}\exp (-\frac{\tau }{{\tau }_{T}})\}$$

where the fractions, *g*_*0i*_ are related to 1/*N*_*i*_ where *N*_*i*_ are the average number of diffusing fluorescent particles in the detection volume), *τ*_D*i*_ are the diffusion times of mRFP-Ras1or Nile Red through the detection volume and the subscripts *1* and *2* represent two different components- one for free (*τ*_*D1*_) and another for membrane bound mRFP-Ras1 or Nile Red (*τ*_*D2*_). *A*_*T*_ and *τ*_*T*_ are the contribution and time-constant of triplet conversion (for Nile Red)/flickering (65 μs for mRFP - fixed). mRFP-Ras1 is localized to the plasma membrane even in wild type cells, as shown by fluorescence microscopy images (Supplementary Fig. 1). However, mRFP-Ras1 may also be present in the cytoplasm and it is possible that the cytoplasmic form could also contribute to the diffusion. It should be noted here that we are using FCS setup with confocal configuration. In this configuration, the typical diffraction limited XY-resolution at image plane is ~285 nm (*r*: radius, i.e., 2*r* ≈ 570 nm) in our FCS setup. However, the typical diameter of *Candida* cells is generally less than 5 μm, while the cell membrane would be much less in width than that. Thus it is highly likely that the diffraction limited spot would encompass regions of both membrane and the cytoplasm and the fluorescence signal comes from both regions. Nevertheless, the diffusion dynamics of cytosolic Ras1 and membrane-bound Ras1 are expected to be drastically different. We have therefore incorporated their contributions to the dynamics in the fitting equation 5 which incorporates two diffusion time-constants, a faster *τ*_*D1*_ and a slower *τ*_*D2*_. The contribution of the free/cytosolic Ras1 to the diffusion time is represented by *τ*_*D1*_. The diffusion time of membrane localized form of Ras1 is represented by *τ*_*D2*_ in that equation. In the wild type strain too, we have both forms. It should be noted that it is the value of τ_*D*2_ that is altered drastically in the mutants, suggesting changes in diffusion of membrane bound Ras1. However *τ*_*D1*_ remains similar and thus, the value of *τ*_*D1*_ has been kept constant keeping its contribution free in the fitting procedure. Thus, *τ*_*D2*_ represents the dynamics of Ras1 bound/near the membrane.

Photobleaching does not seem to be a problem in our case. We can say this because we have recorded all our FCS measurements for 70 seconds over many measurements. Nevertheless, we monitored the fluorescence signal (counts/second) over long time upto 200 seconds; and did not see any major bleaching over 200 s, and nearly no bleaching of signal till ~70 s (our measurement time-window for each FCS-trace). We have not performed any detrending of the data, instead analyzed the FCS-traces using equation-5 directly.

Given the spread in FCS data, we used averages of the normalized autocorrelation curves in all cases to arrive at an average trace which was then fit to the model (equation 5) to obtain average diffusion times and diffusion coefficients of membrane bound Ras1 (Table 1). All correlation data were fitted using equation 5. The raw traces obtained in the different measurements are given in Supplementary Fig. 10. Untransformed cells gave no autocorrelation in FCS measurements (Supplementary Fig. 11).

### Immunostaining of actin filaments

Log phase cells were fixed with formaldehyde, and lyticase treatment was given to the cells. The cells were permeabilized with 0.1% Triton-X 100 for 20 minutes and blocked with 1% BSA for 30 minutes. The cells were washed with PBS and stained with β-actin antibody (A1978 Sigma Aldrich or 13E5 NEB) for staining total actin/and Ras1 antibody (Merck, 05–516) overnight at 4 °C. FITC labelled secondary goat anti mouse antibody (Genei) and TRITC labelled secondary goat anti rabbit antibody (Genei) were used for visualization of actin. For staining of polymerized actin filaments specifically, the cells were stained with rhodamine-phalloidin as described^55^. Cells were spotted on microscopic slides and visualized under Nikon 1X71 confocal microscope or in AIRF, JNU.

### Transcript level analysis using RT-PCR of *HSP90*

RNA isolation and cDNA preparation was done as reported previously^23^. Wild type with the *URA3* marker was taken as the vector control for the *Cagpi19* null since the *URA3* gene is known to affect the transcript levels of many genes involved in hyphal formation^56^. Transcript levels of *HSP90* were quantified using Applied Biosystems RT-PCR machine using HSP90 RT FP and HSP90 RT RP (Supplementary Table II). *GAPDH* was taken as the internal control and transcript levels were measured using GAPDH RT FP and GAPDH RT RP (Supplementary Table II).

### Immunoblotting for detecting phosphorylated Hog1

To compare the activity of Hsp90 in the *Cagpi19* null compared to the WT, the cells were assessed for levels of phosphorylated Hog1 as described previously^25,26^. For immunoblotting, 100 ml culture of the required strain grown in appropriate SD- minimal medium was harvested, washed with sterile water and lysed followed by centrifugation at 2500 g for 10 minutes at 4 °C. The crude lysate obtained was clarified by centrifugation at 16000 g for 30 minutes at 4 °C twice and equal amount of proteins were resolved on a 12% resolving SDS-PAGE. Transfer was set up at 50 V overnight followed by blocking using 4% skimmed milk for 1.5 hours. The blot was then probed with primary antibody (1:500 dilution for Phospho p38 MAPK(Thr180/Tyr182 (9211 S NEB) and 1:2500 dilution for anti-G-6-PDH (A9521 Sigma Aldrich) in TBS + 0.05% Tween-20 at 4 °C overnight followed by washing with TBST (TBS + 0.05% Tween-20). Secondary (goat anti-rabbit) antibody was added at a dilution of 1:5000 in TBS + 0.05% Tween-20 for 2 hours. The blot was developed using ECL kit (G-biosciences).

### Calcein-AM staining

Calcein-AM staining was done to assess the intracellular viscosity by measuring the steady state anisotropy of Calcein in a particular strain. This cytosolic dye is non-fluorescent as such. However, when it goes inside the cytosol of live cells, intracellular esterases cleave the acetoxymethane (AM) moiety to produce Calcein which fluoresces. Log phase cells were taken of 0.5 OD_600 nm_ were taken, washed with PBS and resuspended in 100 μM of Calcein-AM and kept for shaking in dark for 20 minutes. The cells were washed with PBS and resuspended in 1 mL of PBS and proceeded for steady state anisotropy measurements. An excitation of 495 nm (slit width 5 nm) and an emission of 515 nm (slit width 10 nm) was used.

### Sterol estimation

Sterol extraction was done in *Candida albicans* strains by the alcoholic KOH method and estimated by GC-MS as described in^23^.

### Data availability

The datasets generated during and/or analysed during the current study are available from the corresponding author on reasonable request.

## Electronic supplementary material

Supplementary Information
