# Author Correction: Ras hyperactivation versus overexpression: Lessons from Ras dynamics in *Candida albicans*

**DOI:** 10.1038/s41598-019-43265-9

**Published:** 2019-10-15

**Authors:** Vavilala A. Pratyusha, Guiliana Soraya Victoria, Mohammad Firoz Khan, Dominic T. Haokip, Bhawna Yadav, Nibedita Pal, Subhash Chandra Sethi, Priyanka Jain, Sneh Lata Singh, Sobhan Sen, Sneha Sudha Komath

**Affiliations:** 1 School of Life Sciences, Jawaharlal Nehru University, New Delhi, 110067 India; 2 School of Physical Sciences, Jawaharlal Nehru University, New Delhi, 110067 India; 3 Present Address: Post-doctoral fellow, Cell Biology of Mammalian Neurogenesis, Institut Curie, Paris, France; 4 Present Address: Biochemistry and Molecular Pharmacology Department, University of Massachusetts Medical School 55 Lake Ave N, Worcester, 01655 MA USA; 5 Present Address: The Aberdeen Fungal Group, School of Medicine, Medical Science and Nutrition, Institute of Medical Sciences, University of Aberdeen, Aberdeen, AB252ZD UK; 6 Present Address: Department of Chemistry, University of Michigan, Ann Arbor, MI 48109-1055 USA; 7 Present Address: Department of Chemistry, Indian Institute of Technology, Delhi, India

Correction to: *Scientific Reports* 10.1038/s41598-018-23187-8, published online 27 March 2018

This Article contains an error in the X axis of Figure 6g. The correct Figure 6 appears below as Figure 1.

**Figure 1:**
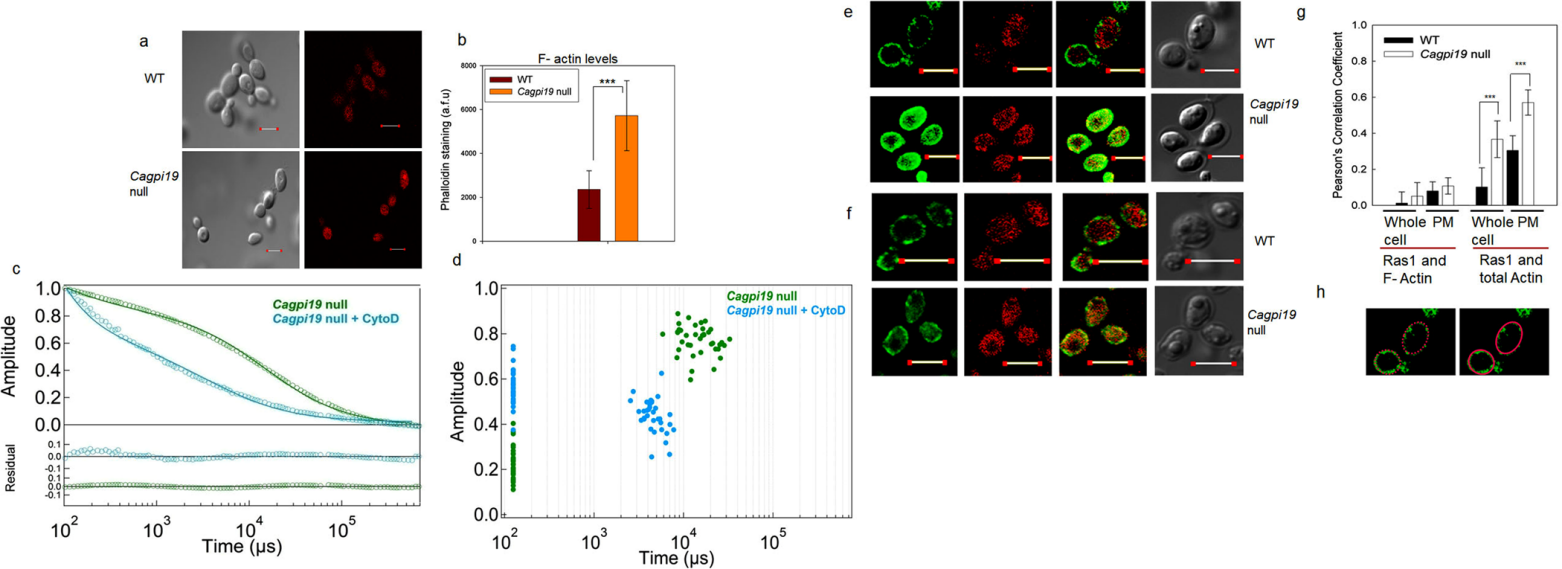
Increased actin polymerization in *Cagpi19* null causes slower Ras1 dynamics. (**a**) Immunostaining of polymerized actin filaments using rhodamine-phalloidin in the wild type (WT) and the *Cagpi19* null mutant. Scale bar corresponds to a distance of 5 μm. (**b**) Quantification of the F-actin levels in the *Cagpi19* null relative to the wild type estimated through rhodamine-phalloidin staining. In each case 40 cells were taken for quantification. Students’ t-test was used to calculate the p value (***p value = 1.27 × 10^−9^). (**c,d**) Average fluorescence autocorrelation curves G(τ) and plot of amplitude versus diffusion times in individual cells after treating the *Cagpi19* null with Cyto D. The data was collected for more than 25 cells in each case. (**e**) Colocalization of Ras1 with the polymerized F-actin in the *Cagpi19* null and the wild type using anti-Ras1 and rhodamine-phalloidin. (**f**) Colocalization of Ras1 with the total β-actin in the *Cagpi19* null and the wild type using anti-Ras1 and anti β-actin antibody. Scale bar corresponds to a distance of 5 μm. (**g**) Quantification of the extent of colocalization of Ras1 with F-actin/total actin using the Pearson’s correlation coefficient. A minimum of 40 cells were taken for quantification in each case. PM indicates plasma membrane. Scale bar corresponds to a distance of 5 μm. Students’ t-test was used to calculate the p value(***p value = 2.25 × 10^−7^ for PM; ***p value 3.98 × 10^−7^ for whole cell). (**h**) Figure showing a representation of the selection of ROI (region of interest) for calculating the Pearson’s correlation coefficient. The left panel shows the selection for calculating the correlation for the plasma membrane. Points all over the plasma membrane were selected for analysis. The right panel shows the selection of the area for calculating the correlation for the whole cell. ROI was selected such that the whole cell was considered for the analysis. Pearson’s correlation coefficient was then calculated using Fluo View software.

