# Supplementary material for: Ras hyperactivation versus overexpression: Lessons from Ras dynamics in Candida albicans
Source: Sci Rep. 2018 Mar 27;8:5248. doi: 10.1038/s41598-018-23187-8 (PMC5869725; doi:10.1038/s41598-018-23187-8)
Supplement: Supplementary file 1 — Supplementary Information [file 41598_2018_23187_MOESM1_ESM.docx]

**Ras hyperactivation versus overexpression: Lessons from Ras dynamics in *Candida albicans*^§^**

**Vavilala A. Pratyusha^1^, Guiliana Soraya Victoria^1#^, Mohammad Firoz Khan^2^, Dominic T Haokip^1ф^, Bhawna Yadav^1₤^, Nibedita Pal^2¶^, Subhash Chandra Sethi^1^, Priyanka Jain^1^, Sneh Lata Singh^1^, Sobhan Sen^2*^ and Sneha Sudha Komath^1*^**

**Supplementary Figures**

**
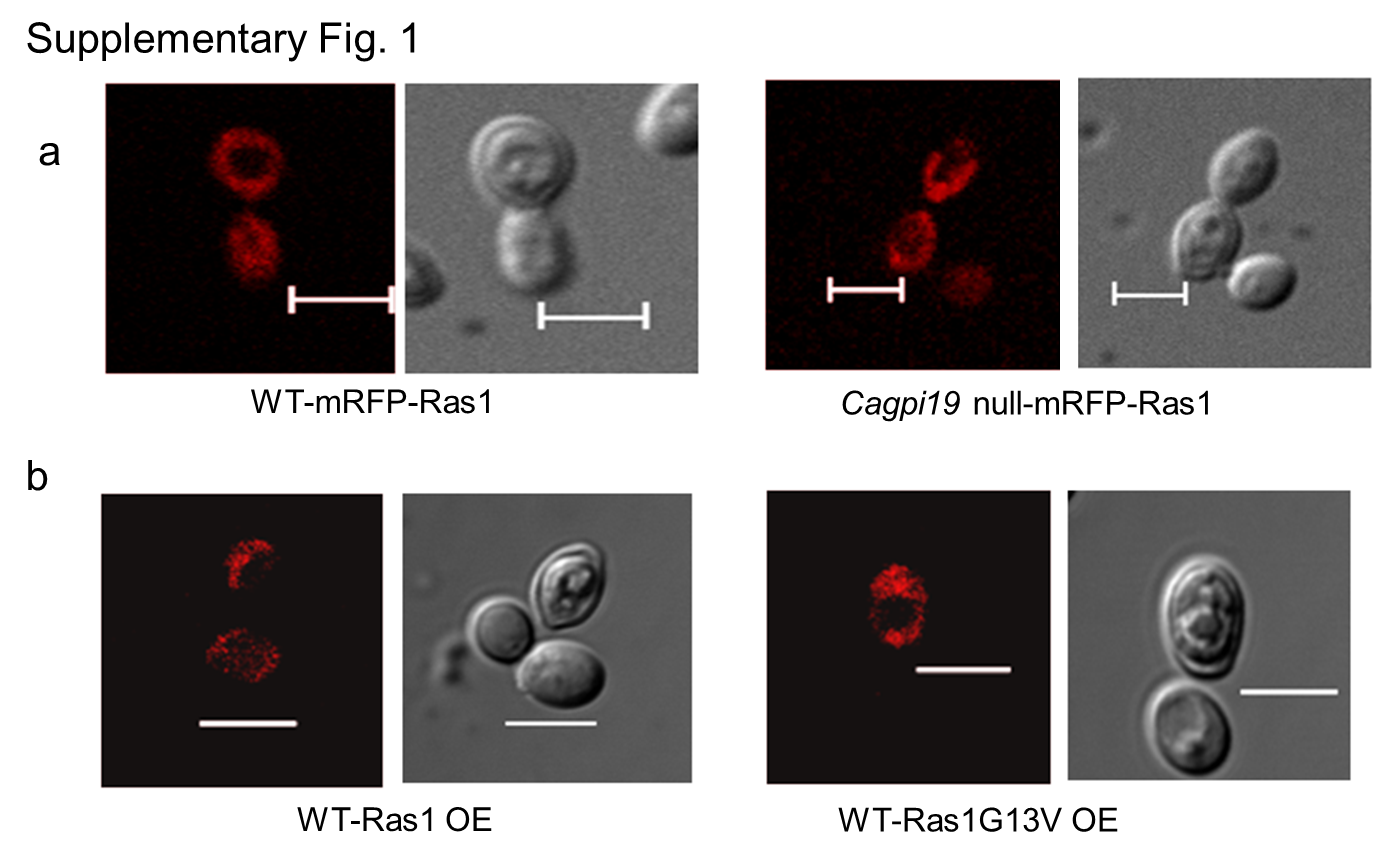
**

**Supplementary Fig. 1: Localization of fluorescently tagged Ras1 and Ras1G13V in the different *C. albicans* strains studied. (a)** Fluorescence microscope images of mRFP-Ras1 in the *C.albicans* wild type (WT) and *Cagpi19* null strains as well as in **(b)** strains over expressing Ras1 or Ras1G13V show that the Ras1 proteins predominantly localize to the plasma membrane. Scale bar corresponds to a distance of 5 µm.


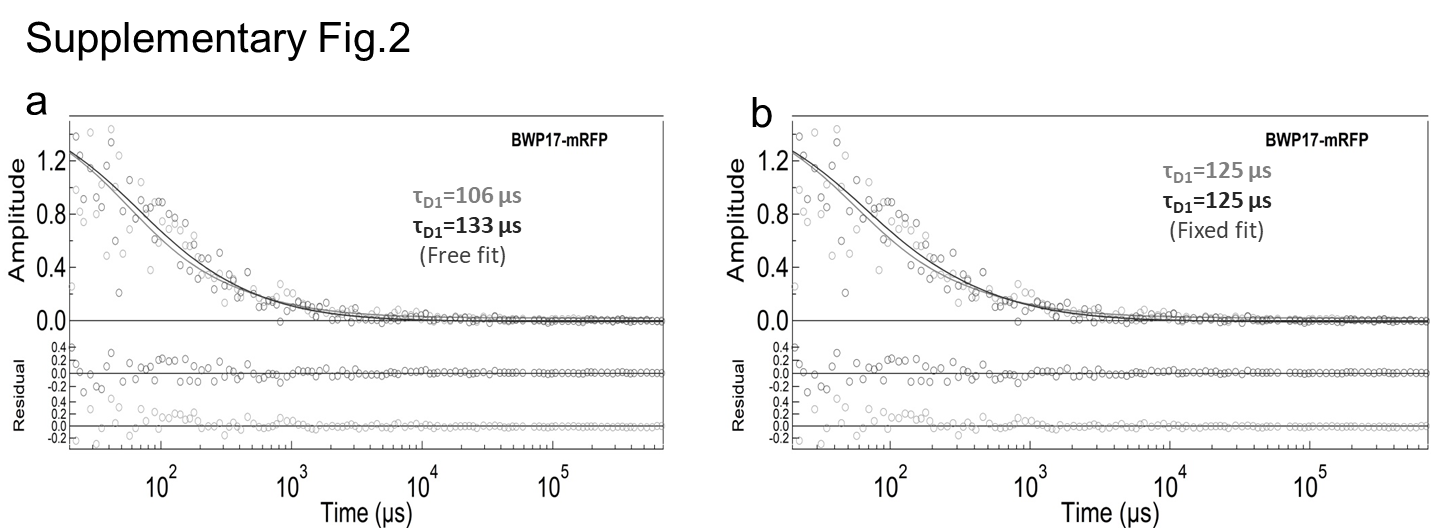


**Supplementary Fig. 2**: **Control FCS data.** **(a)** Autocorrelation curve of free mRFP (transformed into wild type) fixed using a two dimensional one component (2D1C). A diffusion time of 106-133 μs was obtained when a free fitting analysis was done. **(b)** Autocorrelation curve of the same data sets expressing cytosolic free mRFP when *τ_D1_* was fixed at 125 μs.


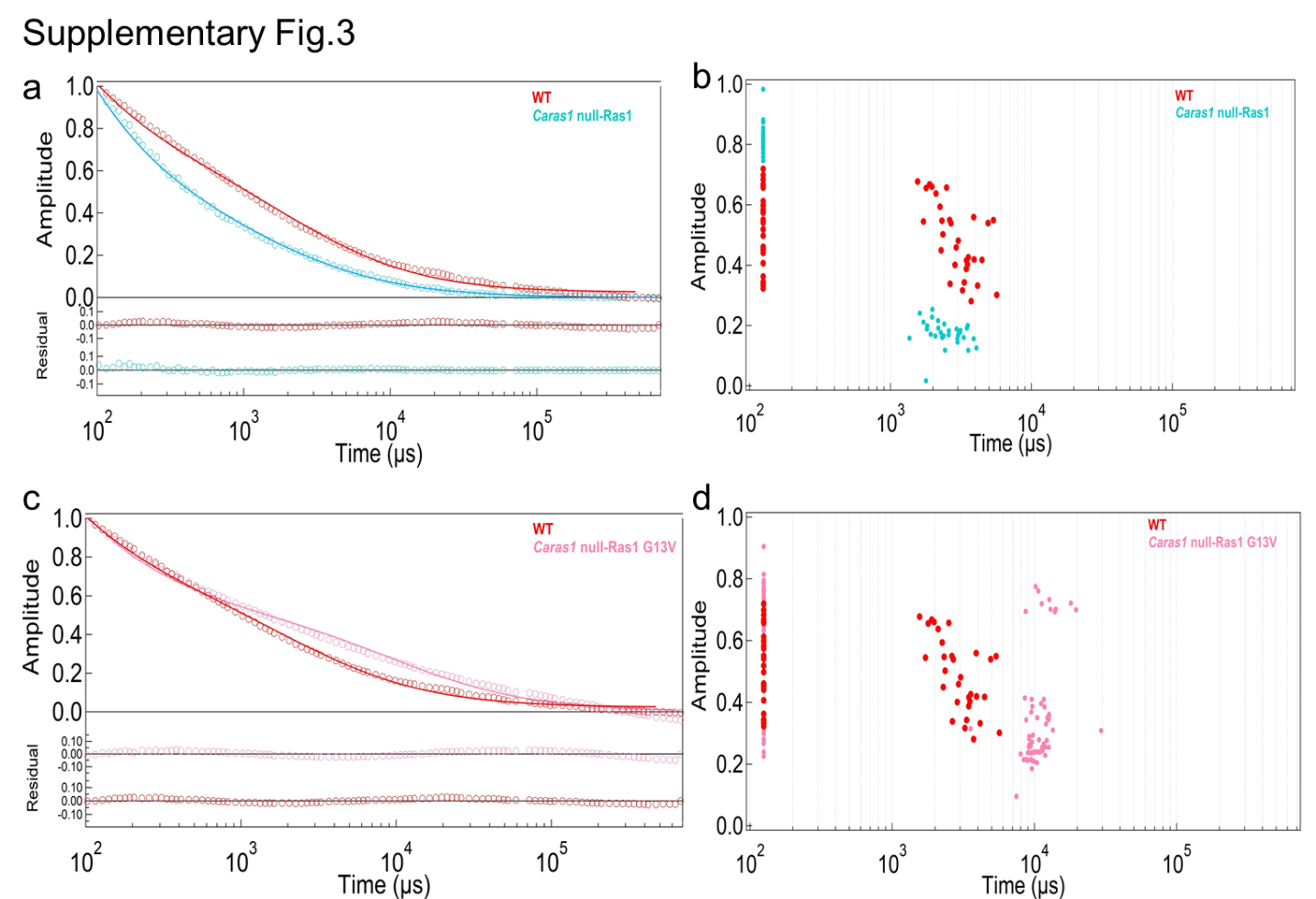


**Supplementary Fig. 3:** **Slower dynamics is seen upon constitutive activation of Ras1 in *Caras1* null**. Average fluorescence autocorrelation, *G*(τ), curves **(a, c)** and plot of amplitude versus diffusion times obtained in individual cells **(b, d)**  for mRFP-Ras1 in wild type (WT) as well as overexpressed states of Ras1 and Ras1G13V in the *Caras1* null mutant*.* Ras1 shows an average diffusion time of 2.24 ms in *Caras1* null-Ras1 and 10.57 ms in *Caras1* null-Ras1 G13V versus 2.63 ms in WT. The data was collected for 35-40 cells in each case.

Supplementary Fig. 4

**
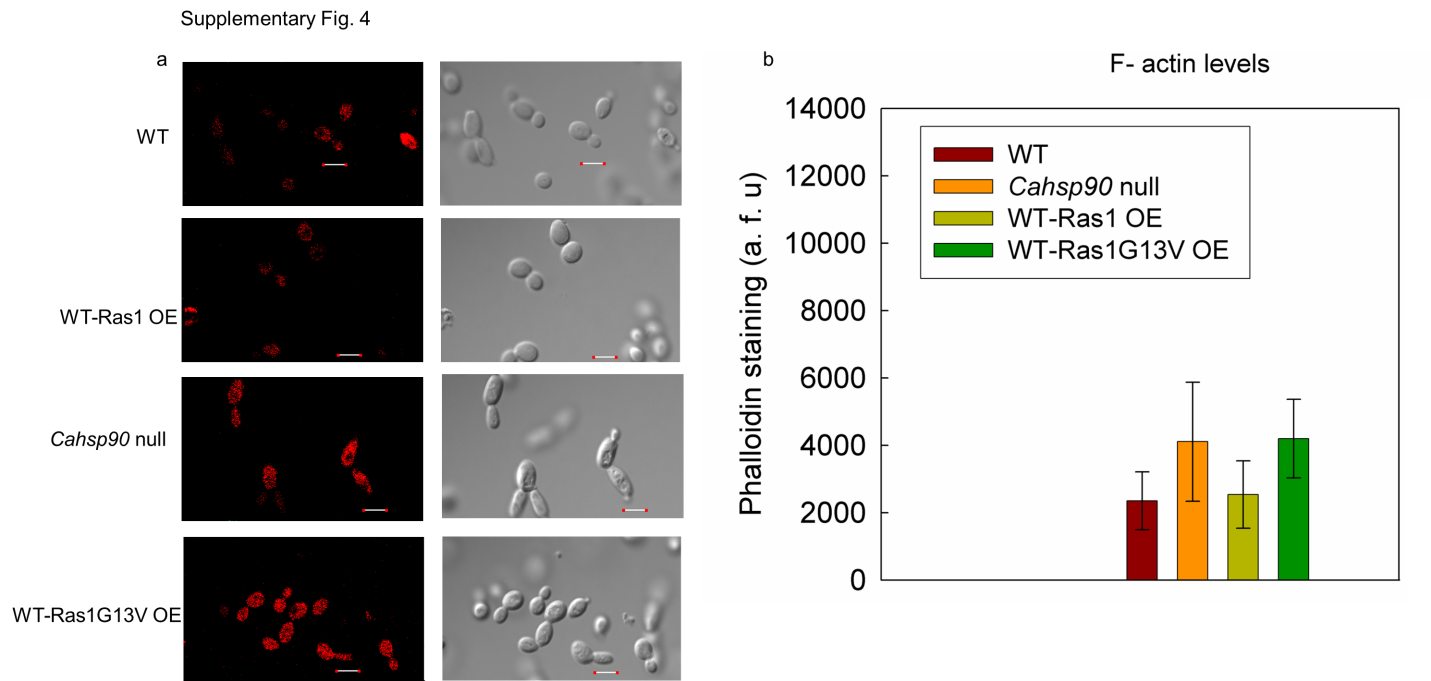
**

**Supplementary Fig. 4: Higher actin polymerization in Ras hyperactivated strains.** **(a)** Phalloidin staining of the indicated strains showing that indeed there is higher actin actin polymerization in strains wherein Ras1 is hyperactivated. **(b)** Quantification of the phalloidin staining indicating that there is a two fold higher actin polymerization in the Ras hyperactivated strains. Quantification was done using Image J.

**
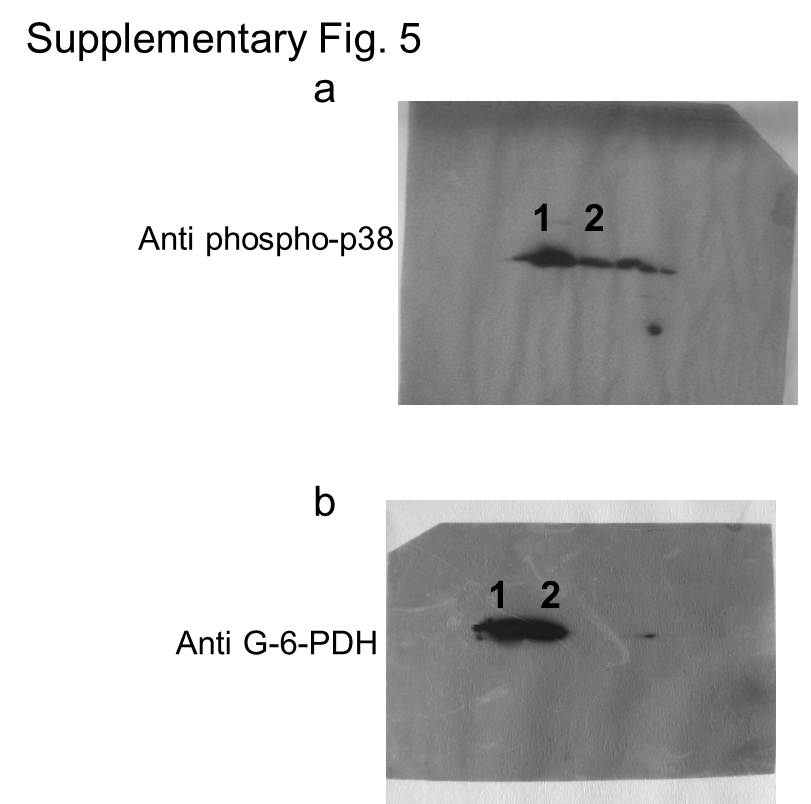
**

**Supplementary Fig. 5: Complete images of the western blots. (a)** Western blot image showing that levels of pHog1 are significantly lower in the *Cagpi19* null than the WT. The bands other than those marked by arrows are of other cell lysates unrelated to this study. **(b)** Image showing equal protein levels of G-6-PDH in WT and *Cagpi19* null. Equal amount of protein (250 μg) was loaded for both the western blots for each strain. Lanes 1 and 2 correspond to WT and *Cagpi19* null respectively in both the western blot images.

**
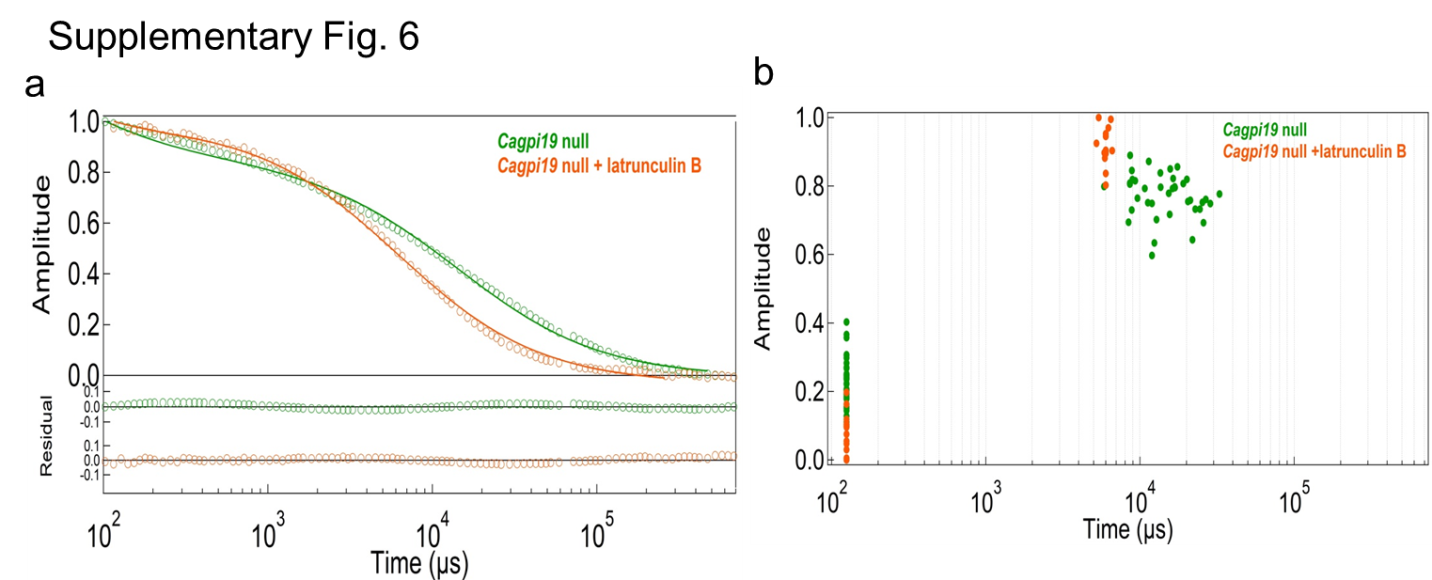
**

**Supplementary Fig. 6: *Cagpi19* null strain treated with the actin polymerization inhibitor latrunculin B shows faster Ras1 dynamics. (a)** Average fluorescence autocorrelation, *G*(τ), curves and **(b)** plot of amplitude versus diffusion times obtained in individual cells for mRFP-Ras1 in *Cagpi19* null and *Cagpi19* null treated with the actin polymerization inhibitor latrunculin B*.* Ras1 shows an average diffusion time of 15.21 ms in *Cagpi19* null and 6.17 ms in *Cagpi19* null. The data was collected for atleast 15-20 cells in each case.

**
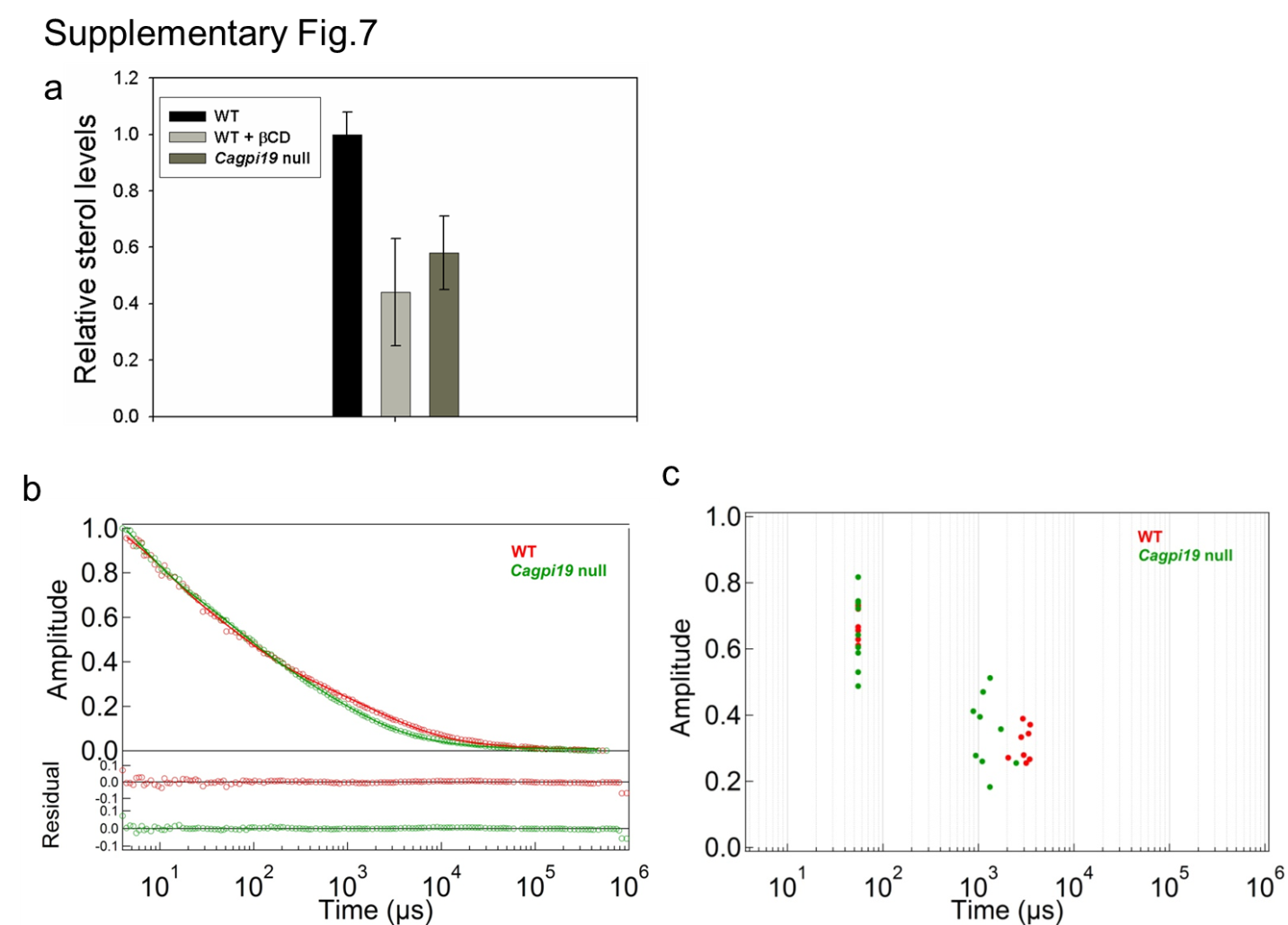
**

**Supplementary Fig. 7: Membrane dynamics of *Cagpi19* null. (a)** Sterol estimation by GC-MS of the indicated *C. albicans* strains. Wild type (WT) cells treated with βCD and the *Cagpi19* null mutant show lower ergosterol levels compared to the wild type untreated. **(b)** Fluorescence autocorrelation curves, *G*(τ) for the dye Nile Red in the cell membranes of *Cagpi19* null compared to the wild type (WT) measured in FCS setup. Solid lines drawn through the data points indicate the two-component 2-D fit as described in the text. **(c**) Plot of amplitude versus diffusion times measured in FCS for the *Cagpi19* null compared to the wild type (WT). The data was collected for 8-10 cells in each case.


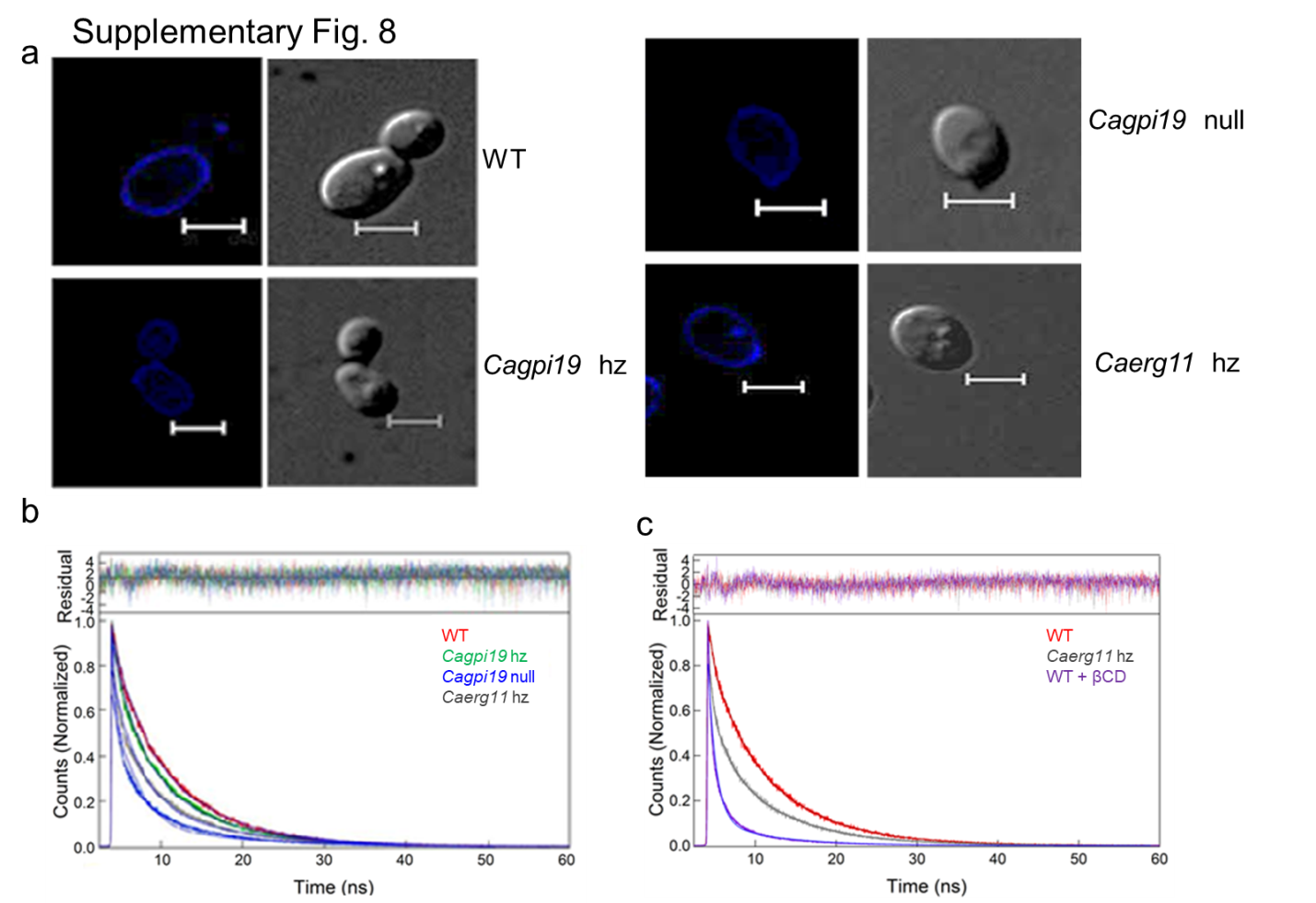


**Supplementary Fig. 8: DPH as a probe to study membrane dynamics. (a)** DPH staining of the indicated *C. albicans* strains to show that it localizes to the plasma membrane. Scale bar corresponds to a distance of 5 µm. **(b)** Normalized fluorescence lifetime decays of DPH in the plasma membranes of the mutants compared to the wild type (WT). Late-log phase cultures were stained with 2 μM of DPH as described in the text and the lifetimes measured in living cells. The graph displays the average decays of five independent experiments. **(c)** Average normalized fluorescence lifetime of DPH in the membrane of the wild type (WT) cells treated with 20 mM βCD as compared to the wild type (WT) control. The decay for the sterol deficient heterozygous *Caerg11* mutant is also shown for comparison. The decays are an average of three independent experiments. The typical instrument response function (IRF) of the instrument was ~85 ps.


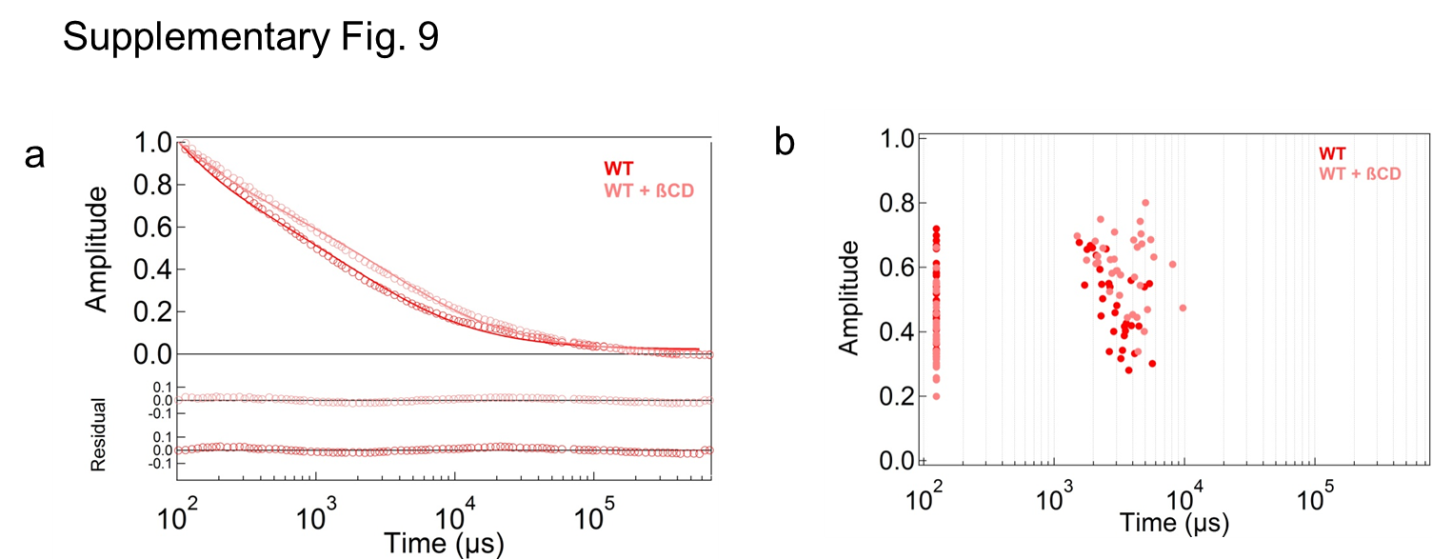


**Supplementary Fig. 9: Sterol deficiency is not responsible for slower Ras1 dynamics in case of Ras1 hyperactivation. (a)** Fluorescence autocorrelation curves *G*(τ) and **(b)** plot of amplitude versus diffusion times after treatment of wild type (WT) cells with βCD. The data was collected for 30-35 cells in each case.


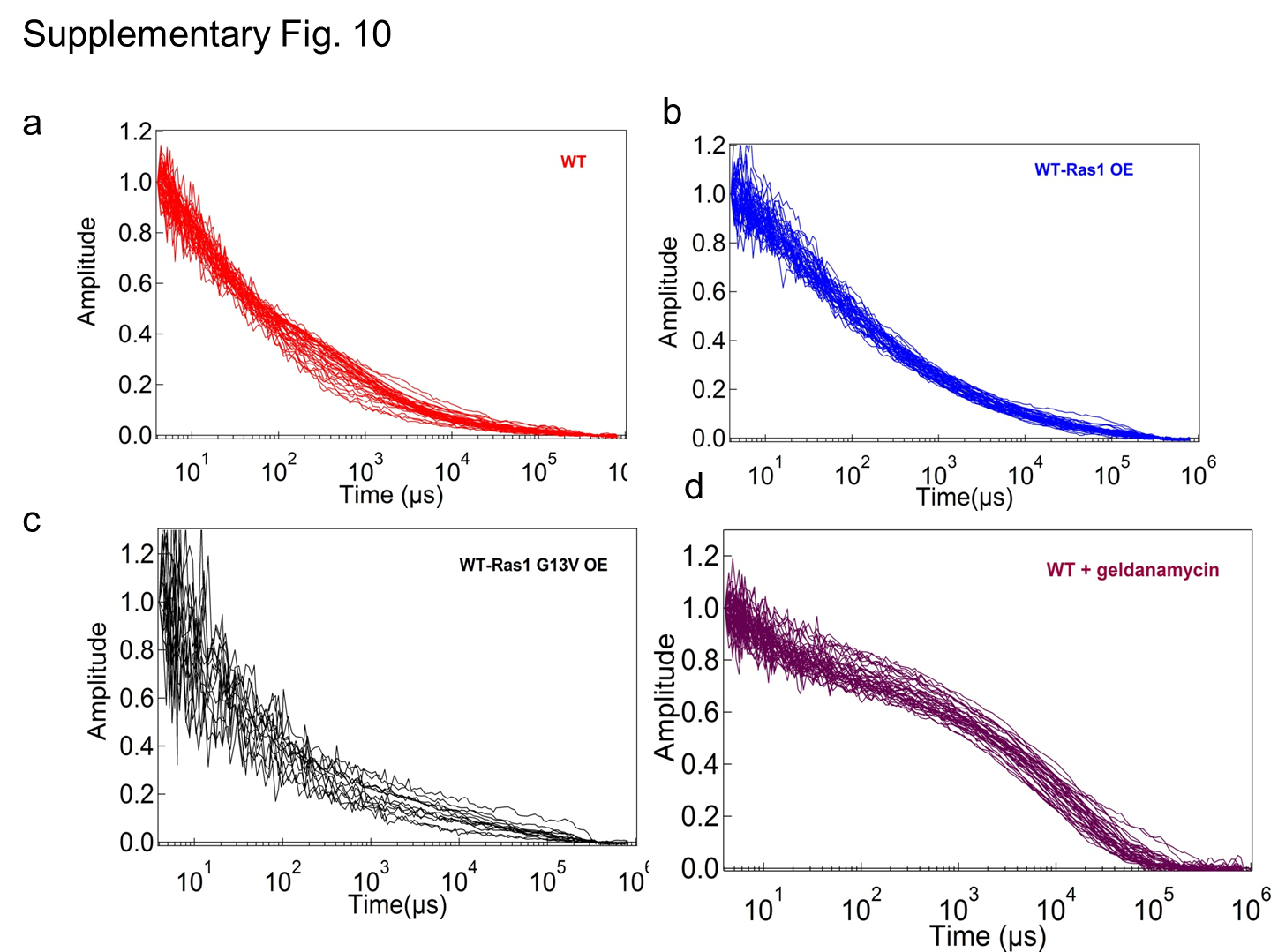


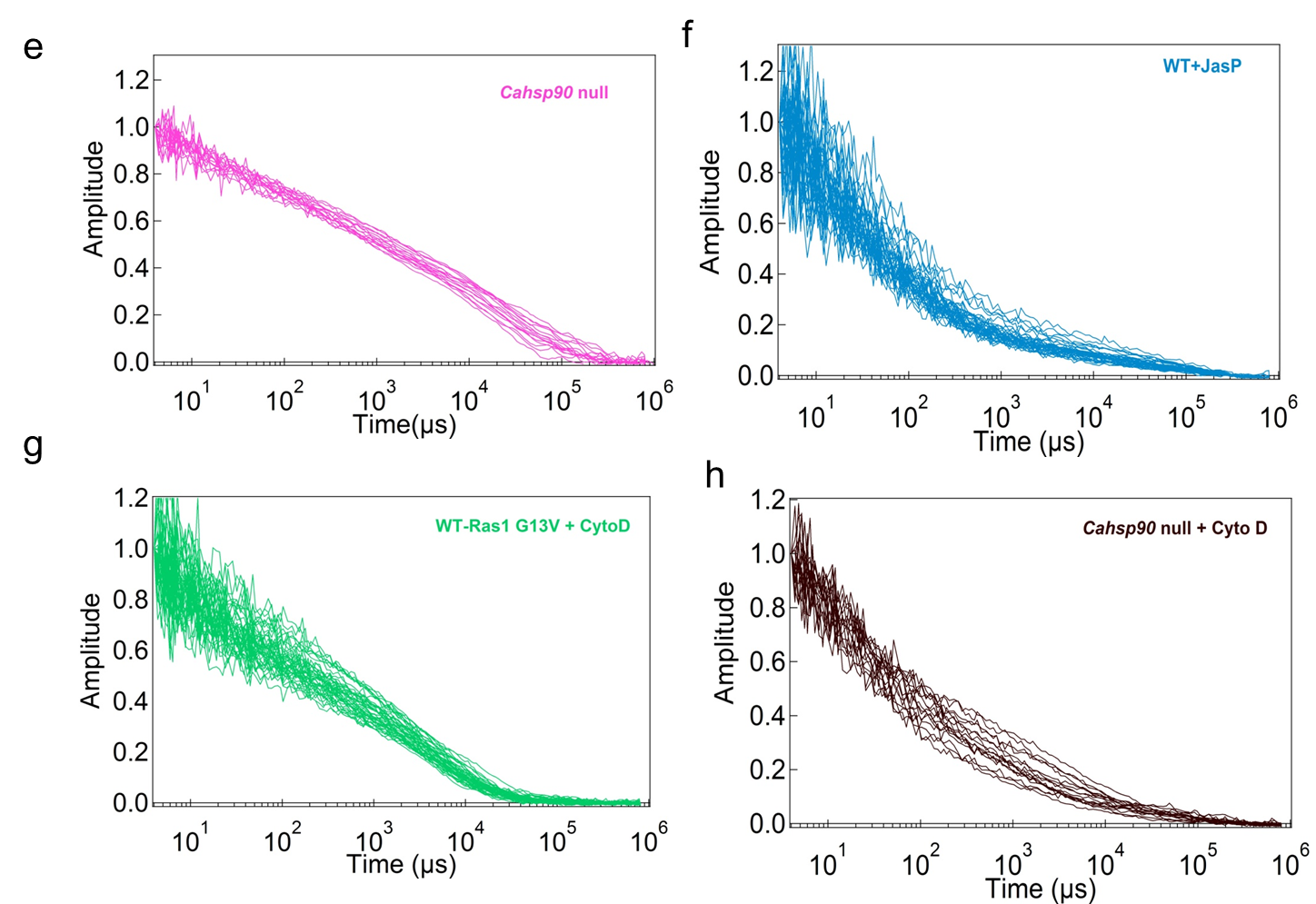

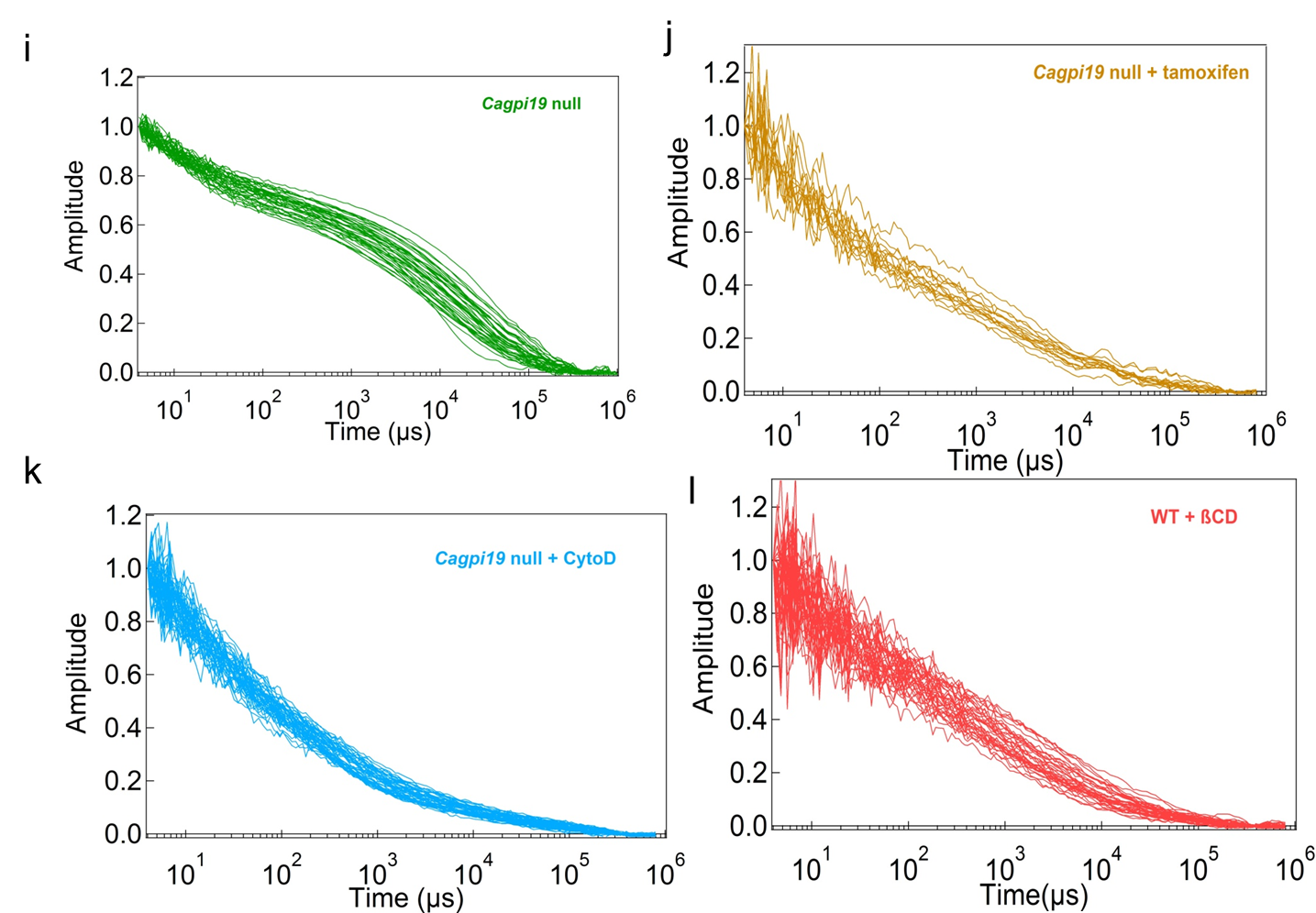

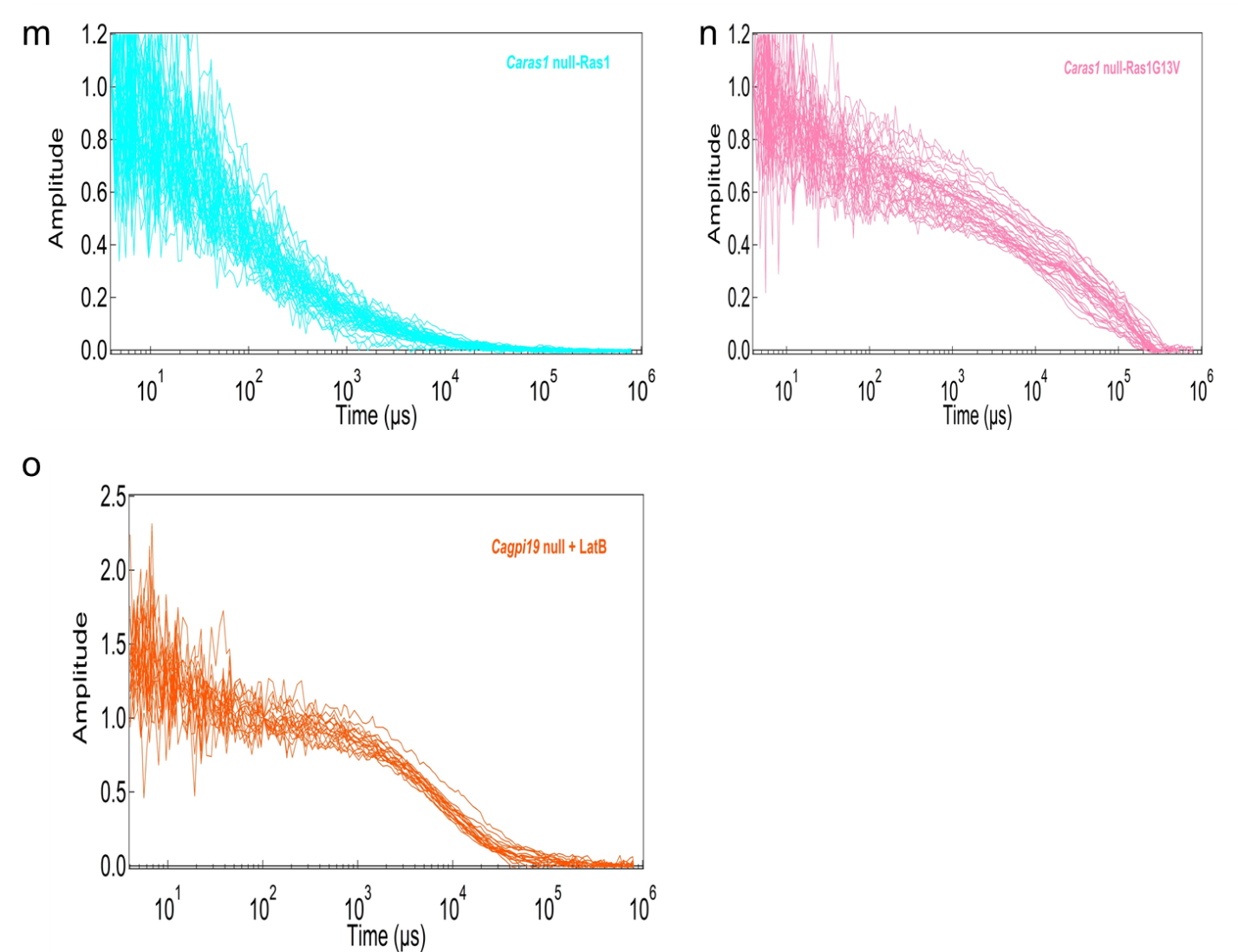


**Supplementary Fig. 10**: **Raw FCS data.** **(a-o)** Raw traces for the FCS measurements of individual cells of the indicated strains are shown in the figure.


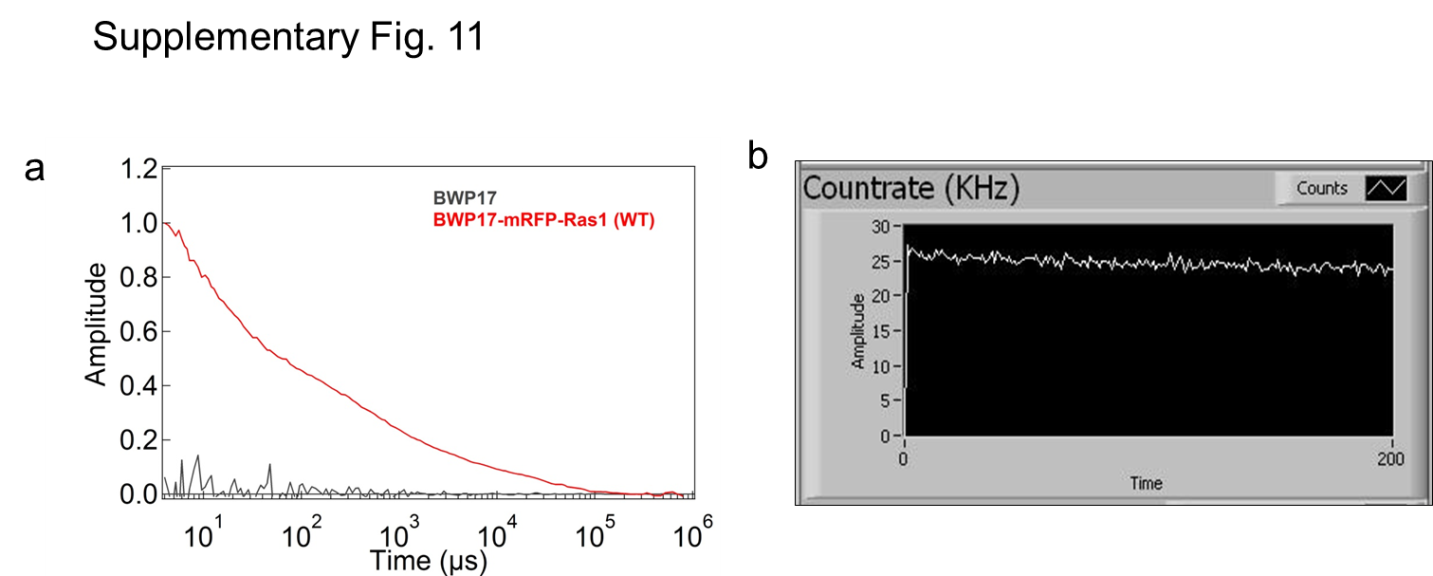


**Supplementary Fig. 11**: **(a)** **Control FCS data.** FCS measurements of untransformed BWP17 cells showing no autocorrelation as opposed to BWP17-mRFP-Ras1 (WT). **(b)** **No photobleaching was observed in the FCS measurements**. Screenshot of FCS measurement showing negligible photobleaching over 200 s and almost no photobleaching over 70 s (over which each FCS trace was measured).

**Supplementary Table I:** Steady state anisotropy (*r_ss_*) and fluorescence lifetimes for DPH in the membranes of heterozygous and null mutants of *CaGPI19*as compared to wild type (WT) cells. Also given for comparison are the fluorescence lifetimes of DPH in the cell membrane of *ERG11* heterozygous mutant and in wild type (WT) cells treated for 1 hr with 20 mM β-CD. Standard deviations between experimental data sets for the lifetime measurements ranged between ± 5% of the average in all cases.

| **Strain** | ***r_ss_*** | ***f_1_* (%)** | ***τ_1_* (ns)** | ***f_2_* (%)** | ***τ_2_* (ns)** | ***f_3_* (%)** | ***τ_3­_* (ns)** | ***‹τ›***  **(ns)** | **χ^2^** |
| --- | --- | --- | --- | --- | --- | --- | --- | --- | --- |
| Wild type (WT) | 0.178±0.005 | 26 | 0.310 | 14 | 2.12 | 60 | 7.89 | 5.11 | 1.07 |
| *CaGPI19* heterozygote | 0.172±0.008 | 25 | 0.266 | 25 | 1.42 | 50 | 7.76 | 4.30 | 1.02 |
| *Cagpi19* null | 0.147±0.005 | 55 | 0.167 | 26 | 1.48 | 19 | 7.95 | 1.99 | 1.01 |
| *ERG11* heterozygote | 0.148±0.003 | 47 | 0.178 | 22 | 1.67 | 31 | 7.92 | 2.91 | 1.01 |
| Wild type (WT) + βCD | 0.137±0.005 | 63 | 0.200 | 30 | 1.39 | 7 | 7.10 | 1.04 | 1.05 |

**Supplementary Table II**

List of primers used in this study.

| **Primer** | **Sequence (5'-3')** |
| --- | --- |
| RFPFF FP | GCGCCCGGGATGGTTTCAAAAGGTGAAGAA |
| RFPFF RP | GCGGGTACCTTCTGATGAAGCTTCCCAACC |
| RFPSF FP | GCGGAGCTCGATTATTTAAAATTGTCATTT |
| RFPSF RP | GCGGGCGCCTTATTTATATAATTCATCCAT |
| Ras-RFPFP | AAAAAGAAACCCCGGGCAAACACAAATTCATATCCACACATATACATACCATGGTTTCAAAAGGTGAAGAAGATAATAT |
| Ras-RFPRP | GATTTACCAACACCACCACCTCCAACAACAACTAATTTATATTCTCTCAATTTATATAATTCATCCATACCACCAGTTG |
| Ras1 FP | GCGAAGCTTATGTTGAGAGAATATAAATTA |
| Ras1 RP | GCGGCTAGCTCAAACAATAACACAACATCC |
| RFP FP | GCGCTGCAGATGGTTTCAAAAGGTGAAGAA |
| RFP RP | AAACTGCAGTTTATATAATTCATCCATACC |
| Ras1G13V FP | AAATTAGTTGTTGTTGGAGGTGTTGGTGTT |
| Ras1G13V RP | TAAAGCGGATTTACCAACACCAACACCTCC |
| CaHSP90 HISI FP | ATGGCTGACGCAAAAGTTGAAACTCACGAATTCACTGCTGAGATCTCTCAGTTGAACCGGGGATCCTGGAGGATGAG |
| CaHSP90 HISI RP | TTAATCAACTTCTTCCATAGCAGATTCTCCAGCTGGTTCGTCAGTTGAGGCAGTAAACGGAATATTTATGAGAAACT |
| CaHSP90 FP | GCGAAGCTTATGGCTGACGCAAAAGTTGAA |
| CaHSP90 RP | GCGGCTAGCTTAATCAACTTCTTCCATAGC |
| CaHSP90 NULL FP | GTAGAAAAAAACATTATAGAATGTTCTTTTTGGTTCTATAGAATTCCATCAGAAAATCTAGAAGGACCACCTTTGATTG |
| CaHSP90 NULL RP | AAGACATCAACTGAGAGATCTCAGCAGTGAATTCGTGAGTTTCAACTTTTGCGTCAGCCATTTTAATAAACGCGGATCC |
| HRAS FP | GCGAGATCTATGACGGAATATAAGCTGGTG |
| HRAS RP | GCGAAGCTTTCAGGAGAGCACACACTT |
| HRAS G12V FP | AAG CTG GTG GTG GTG GGC GCC GTC GGT GTG |
| HRAS G12V RP | CAG CGC ACT CTT GCC CAC ACC GAC GGC GCC |
| HSP90 RT FP | AAGTGCTGGTGCTGACGTTT |
| HSP90 RT RP | ACCACCAGCGTTAGATTCCC |
| GAPDH RT FP | CAGCTATCAAGAAAGCTTCTG |
| GAPDH RT RP | GATGAGTAGCTTGAACCCAA |
